# Optically activated, customizable, excitable cells

**DOI:** 10.1371/journal.pone.0229051

**Published:** 2020-12-30

**Authors:** Merrilee Thomas, Thomas E. Hughes

**Affiliations:** 1 Department of Neuroscience and Cell Biology, Montana State University, Bozeman, Montana, United States of America; 2 Montana Molecular, Bozeman, Montana, United States of America; Indiana University School of Medicine, UNITED STATES

## Abstract

Genetically encoded fluorescent biosensors are powerful tools for studying complex signaling in the nervous system, and now both Ca^2+^ and voltage sensors are available to study the signaling behavior of entire neural circuits. There is a pressing need for improved sensors, but improving them is challenging because testing them involves a low throughput, labor-intensive processes. Our goal was to create synthetic, excitable cells that can be activated with brief pulses of blue light and serve as a medium throughput platform for screening the next generation of sensors. In this live cell system, blue light activates an adenylyl cyclase enzyme (bPAC) that increases intracellular cAMP (Stierl M et al. 2011). In turn, the cAMP opens a cAMP-gated ion channel. This produces slow, whole-cell Ca^2+^ transients and voltage changes. To increase the speed of these transients, we add the inwardly rectifying potassium channel Kir2.1, the bacterial voltage-gated sodium channel NAVROSD, and Connexin-43. The result is a highly reproducible, medium-throughput, live cell system that can be used to screen voltage and Ca^2+^ sensors.

## Introduction

### Why are biosensors important?

Genetically encoded, fluorescent biosensors are powerful tools for studying cell signaling in real-time [1, 2]. They are minimally invasive, and since they are genetically encoded their expression can be targeted to specific cell types and tissues [3–5]. However, the short wavelengths of light needed to image green biosensors can heat and damage the brain [6]. There is a need for better biosensors that emit red [7] or near-infrared [8] light because these longer wavelengths enable investigators to image deeper into thick tissues. Furthermore, increases in the signal to noise ratio of these sensors empowers scientists to collect more information from ever increasing numbers of cells [9, 10]. An additional benefit of brighter sensors with high SNR is that they can be detected at lower expression levels. Higher levels of Ca^2+^ sensor expression levels can lead to epileptiform activity [11].

A proven approach to expanding the fluorescent biosensor toolbox involves screening thousands of prototypes, evolving for optimal function [12–15]. One way this has been done is expressing mutagenized libraries of Ca^2+^ sensors in *E*. *coli*. One study used a microfluidic sorter (*μ*FACs) where it was possible to screen up to 10^6^ colonies per round of evolution in *E*. *coli* [7, 16]. A more recent study optimized sensors by picking *E*. *coli* expressing sensors with the greatest fluorescence intensity, followed by a second screen of bacterial lysates in Ca^2+^ and Ca^2+^ free buffers [17]. A drawback to this approach is that a sensor cannot be screened for its kinetics, there are only two steady states.

Mammalian excitable cells can be used to screen activity biosensors. However, screening in primary cultures of differentiated, excitable cells is time consuming, expensive, and low throughput [18]. Another approach is to create excitable cells that can be easily transfected and studied. One of the first attempts at creating *de novo* excitable cells expressed the rat brain IIA Na^+^ channel and the Drosophila Shaker K^+^ channel in CHO cells via vaccinia virus [19]. Additionally, one study stably expressed three ion channels tagged with fluorescent markers, Kir2.1, Nav1.5, and Cx-43 in HEK293 cells. They found they could create fast uniform action potentials but could not be used to test biosensors because of the fluorescent markers used [20].

There have been efforts to test and screen voltage sensors in HEK293 cells that combine Kir2.1 and a voltage-gated sodium channel (NAV1.3) with field stimulation to produce rhythmic depolarization and hyperpolarization in HEK cells [13, 21, 22]. Another option is to use a stable cell line that expresses Kir2.1, Nav1.7, and CheRiff, a channelrhodopsin. This can be photoactivated thereby creating a system that produces oscillating membrane voltage [23] without field stimulation. However, there are problems with stable cell lines expressing ion channels such as genetic drift and gene silencing. Often stable cell lines will regulate the expression of exogenous genes, and mutations in the exogenous genes will occur after several cell culture passages [24, 25]. In addition, the level of expression of each of the channels cannot be easily changed and currently, only wavelengths outside of the rhodopsin's excitation spectrum can be used for screening.

Our goal was to create a screening platform for biosensors that is consistent, reliable, and capable of quickly screening thousands of prototypes. We packaged all of the components in the BacMam viral delivery system, which made it possible to optimize the relative expression levels. BacMam viral delivery is a common tool in high throughput drug discovery because of the consistency of expression, the shelf stability of the virus, and the broad tropism of the VSVG-pseudotyped baculovirus [26]. We first created a minimal cellular system with a light-activated enzyme bPAC to establish a “Kuhl” cell. Next, we added a cAMP-gated ion channel, either a CNG or HCN channel, to produce “Kuhl-C, and Kuhl-H” cells respectively. We then added additional channels and proteins to tune the responses. To improve the responses we added the Kir2.1 potassium channel, to create Kuhl-CK or Kuhl-HK cells. To speed up the action potentials we added the bacterial sodium channel NavD to design Kuhl-CKNa and Kuhl-HKNa cells. Finally, we found that connexin-43 improved the coordination of the signaling, producing Kuhl-CKNaCx and Kuhl-HKNaCx cells.

## Results

### Establishing an optical actuator

How can we activate a biosynthetic cell line using light? To develop an optogenetically controlled screen, we chose to use bPAC (blue photo-activated cyclase), a blue light activated adenylyl cyclase from the soil bacteria *Beggiatoia* [27]. In theory, coupling a light activated enzyme to large conductance channels should produce larger whole cell currents with less protein expression than the current optogenetic approaches that rely upon channel rhodopsins. The bPAC enzyme is activated with 480 nm light and converts ATP into cyclic adenosine monophosphate (cAMP). We began by examining whether blue light stimulation can produce a measurable change in cAMP levels in bPAC-expressing HEK293 cells as seen with R-cADDis, a red fluorescent cAMP sensor [28].

**Fig 1:**
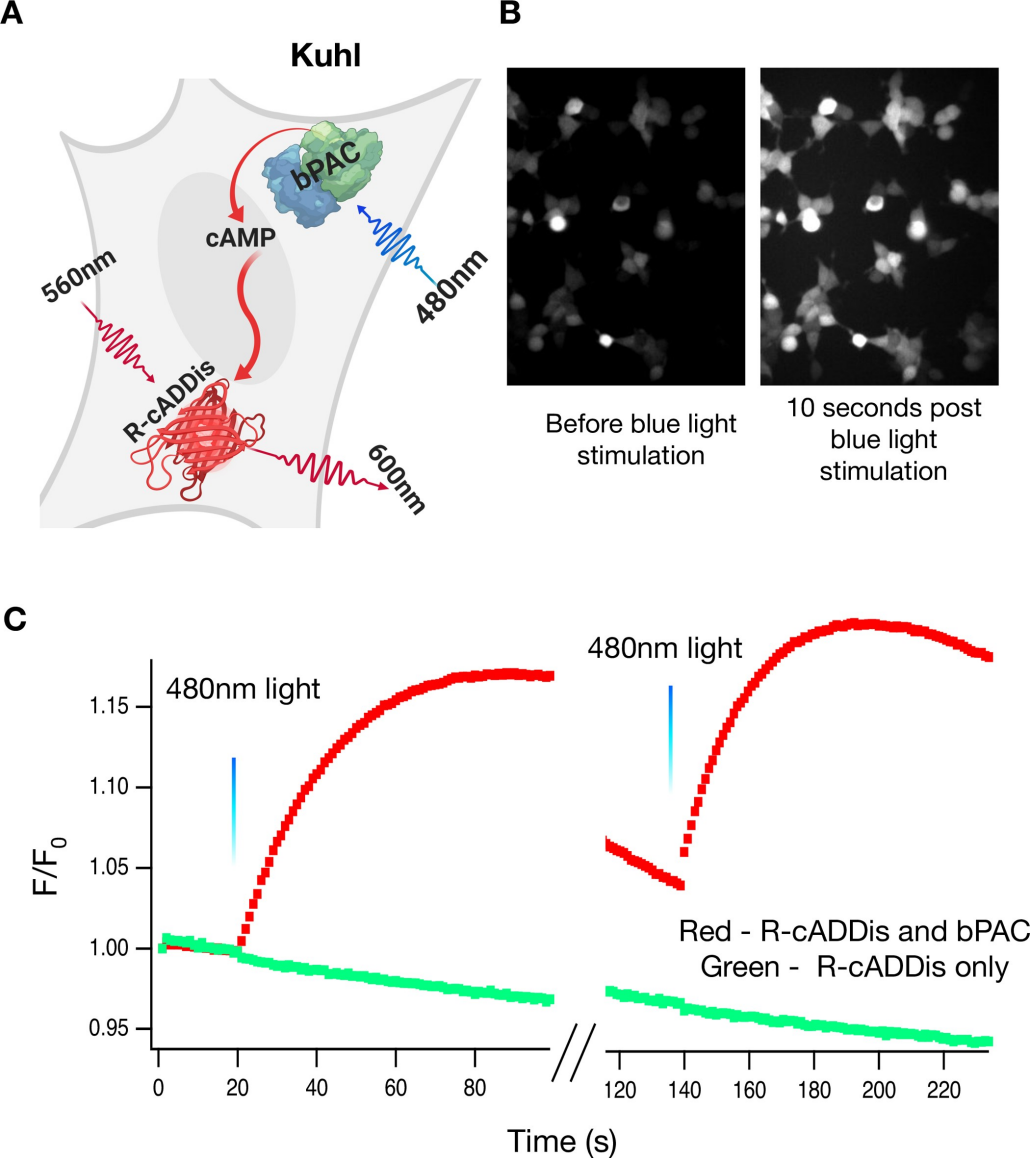
20ms of blue light can repeatedly activate bPAC. A) HEK-293 cells were transduced with bPAC (the actuator) and R-cADDis, the red fluorescence cAMP sensor (S1 Table). B) The cells were illuminated with 20ms of 480nm light. This activation of bPAC increases cAMP levels which increases R-cADDis fluorescence. Brightness and contrast are the same for both images. C) The traces are a discontinuous recording of R-cADDis in the same cell (100s on, 20s off, 100s on), illustrating that the bpac response can be activated multiple times. Twenty milliseconds of blue light at 20s and 140s activates bPAC which produces bursts of cAMP for 100s. This is a stereotyped response that is repeatable with multiple doses of blue light.

We transduced HEK293 cells with BacMam viruses to co-express bPAC and R-cADDis (S1 Table). The following day, we stimulated the cells with 20 milliseconds of blue light and then collected images continuously with 561 nm excitation light that does not activate bPAC (Fig 1A). Upon the blue light stimulation, the R-cADDis red fluorescence increases (Fig 1B). cAMP levels within the cell rise over the time frame of ~100 seconds (Fig 1C). Control cells with no bPAC did not show an increase in red fluorescence. The system response is remarkably reproducible and the same cells can be repeatedly stimulated. Indeed the only limitation is that too much blue light given over a short time will create too much cAMP and saturate the sensor. The slow decrease in fluorescence of R-cADDis is most likely due to the phosphodiesterases present in the cell which work to eliminate cAMP [29].

### Coupling the actuator with an ion channel

To couple cAMP production with channel activation, we expressed a cyclic nucleotide-gated ion channel. We tried both the rat olfactory CNG and the human heart HCN2 channel (S1 Table). The rat olfactory CNG channel is normally gated by cyclic guanosine monophosphate (cGMP) [30]. However, several mutations (*δ*61–90/C460W/E583M) can be introduced [31] that renders it sensitive to cAMP. In theory, this mutant channel should couple bPAC activation to a current that depolarizes the cell, creating Kuhl-C cells. To create Kuhl-H cells, we expressed the human HCN2 pacemaker channel which is also cAMP-gated and is hyperpolarization-activated (S1 Table) [32]. Once the cell is hyperpolarized, the HCN2 channel can open when bound to cAMP.

**Fig 2:**
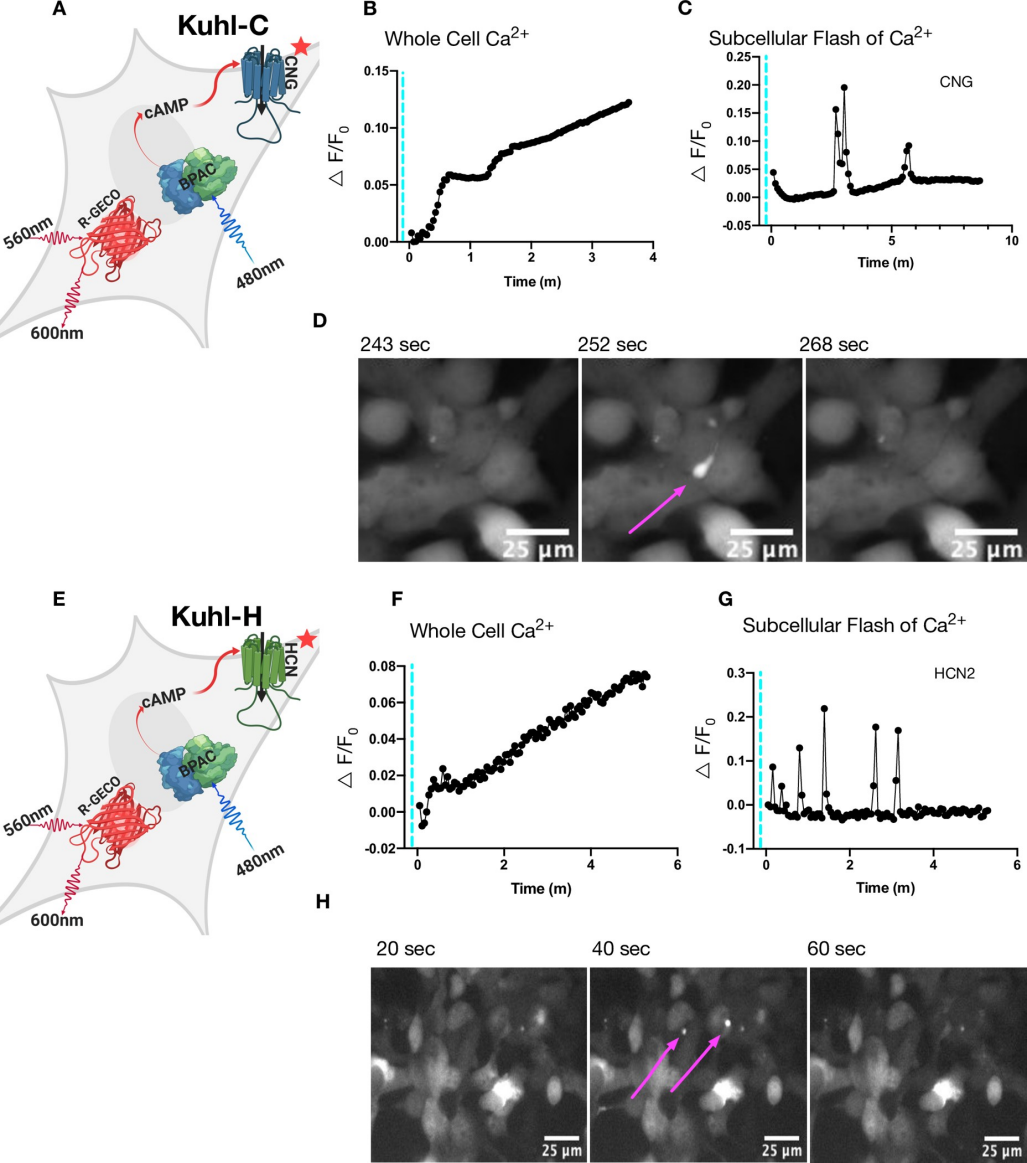
Blue light activation of bPAC slowly increases whole-cell Ca^2+^ when CNG or HCN2 is expressed. Brief pulses of blue light produced two types of increases in Ca^2+^ fluorescence changes: a slow whole-cell Ca^2+^ increase and bright subcellular flashes of Ca^2+^. A) Cartoon depicting all the components in the Kuhl-C cells. Blue light stimulation of bPAC produces cAMP that should in turn open a cAMP gated CNG (delta61–90/C460W/E583M) channel. B) The black trace is the R-GECO1 response to 30 ms of blue light activation in Kuhl-C cells. C) The black trace depicts small brief subcellular flashes of Ca^2+^ that can be seen in cells with CNG and bPAC. D) The localized subcellular flash of Ca^2+^ seen in the Kuhl-C cell system in a series of three frames at 243s, 252s, 268s. E) Cartoon depicting all the components in the Kuhl-H cells. F) The R-GECO1 response to 30 ms of blue light activation in Kuhl-H cells. G) Small brief subcellular flashes of Ca^2+^ that can be seen in Kuhl-H cells. H) The localized subcellular flash of Ca^2+^ seen in Kuhl-H cells at 20s, 40s, 60s.

We activated the Kuhl-C or Kuhl-H cells with blue light to increase cAMP. Fig 2 illustrates that 20ms of blue light activation of bPAC, produces an increase in the red fluorescence of Ca2^+^sensor R-GECO1 [7], indicating a gradual increase in whole-cell Ca^2+^ over several minutes. Interestingly, blue light stimulation also produced bright subcellular flashes of fluorescence in both the Kuhl-C and Kuhl-H cells. The source of this Ca^2+^ is not clear, but one possibility is that increases in cAMP could be causing brief IP_3_ channel opening, or perhaps mitochondrial release [33] (Fig 2C and 2G). These small flashes of Ca^2+^ last ~20 seconds (Fig 2D and 2H and S1 Movie). The control cells without bPAC showed no such responses (S1 Fig).

### Addition of Kir2.1 coupled with bPAC’s cAMP activity leads to fluctuating whole-cell Ca^2+^ transients

The co-expression of bPAC and CNG, or HCN2, produced reliable Ca^2+^ transients, but these were long-lived events with slow kinetics. Introducing a voltage-regulated inward-rectifying potassium channel should increase the speed and amplitude of these transients by creating a greater driving force on Ca^2+^ to enter the cell. Wild type HEK293 cells have a resting membrane potential of only -25 mV, but Kir2.1 expression shifts their resting potential to -70mV [20, 34–36].

**Fig 3:**
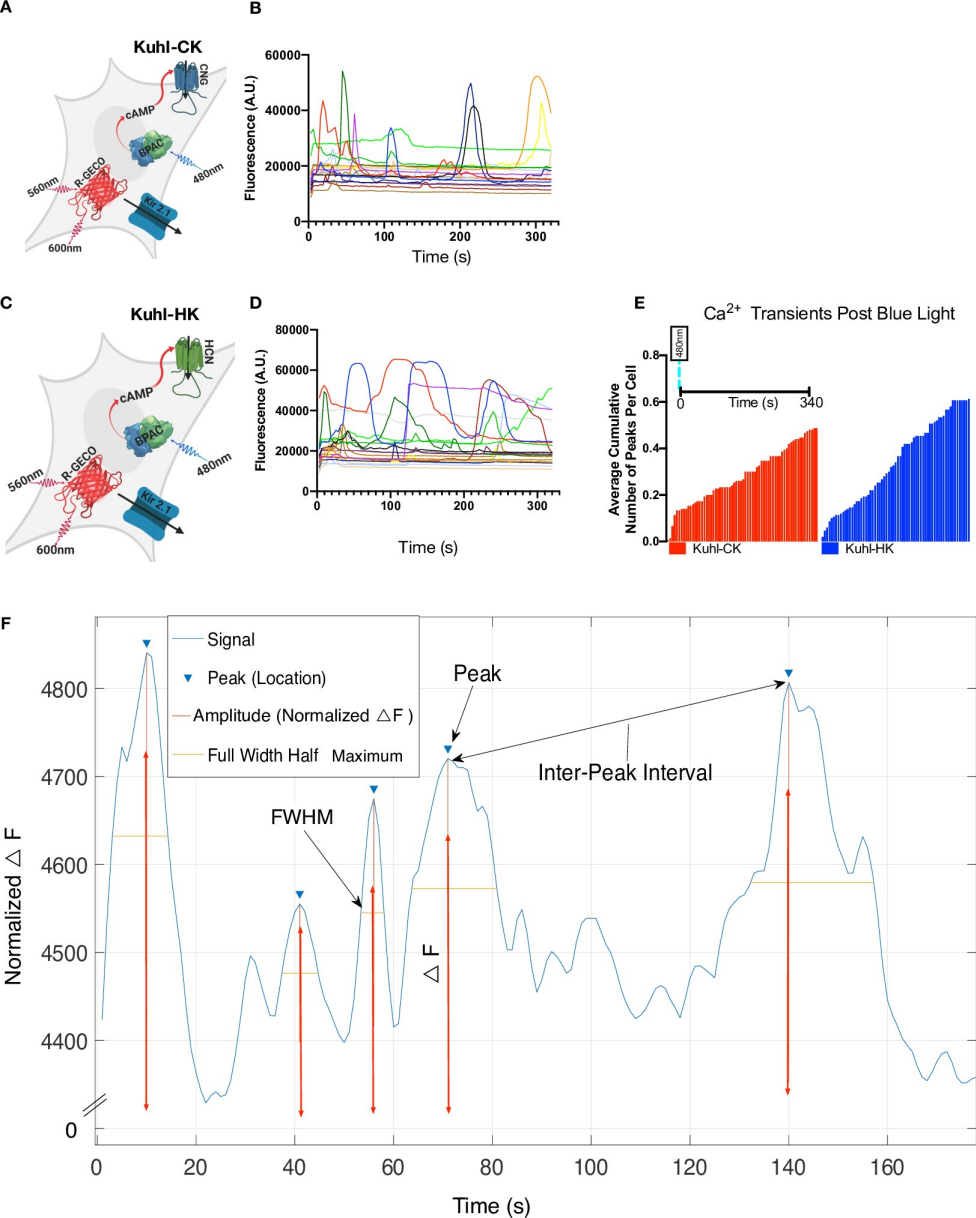
Addition of Kir2.1 leads to fluctuating whole-cell Ca^2+^ transients in the Kuhl-C/HK system. A) Cartoon depicting all the components transduced into the experimental Kuhl-CK cell system. Blue light stimulates bPAC which will produce cAMP that opens the CNG channel, and Kir2.1 should hyperpolarize the cells, creating a greater driving force on Ca^2+^ to enter the cell. B) R-GECO1 fluorescence response to 20ms of blue light in Kuhl-CK. C) Cartoon depicting the experimental Kuhl-HK cell system. D) 20ms of blue light stimulating Kuhl-HK cells. Each of the traces is collected from a single cell. E) Average cumulative number of Ca^2+^ peaks in Kuhl-HK and Kuhl-CK system (all peaks detected across 150 cells for each condition over 340s). Kuhl-HK cells have a higher average cumulative number of peaks per cell in 340s. Each cell has a unique fluctuating Ca^2+^ transient response after the blue light stimulation of bPAC. F) Diagram of the MATLAB analysis of the Ca^2+^ transients. Peak intensity, the full width of the peak at half maximum (FWHM), and interpeak intervals were measured for every event that exceeded our threshold of an SNR >26.

We co-expressed Kir2.1, bPAC, R-GECO1, and a cyclic nucleotide-gated channel in HEK293 cells to produce Kuhl-CK and Kuhl-HK cells (S1 Table). In the Kuhl-C/HK cells, there is some degree of spontaneous activity, meaning Ca^2+^ fluorescence fluctuations, before blue light stimulation. There are Ca^2+^ transients that occur in 85% of the cells, but the activity is largely uncoordinated. Each cell has its own “signature” change in Ca^2+^ levels, as seen in the R-GECO1 fluorescence traces (Fig 3B). Ca^2+^ transients can be seen for up to 30 minutes following the blue light stimulus (S2 Fig). The histogram shows the average cumulative number of fluorescence peaks per cell over 340s (Fig 3E). The time scale is the same for each bar within the histogram. HCN2 produces a slightly greater cumulative average frequency of Ca^2+^ fluorescence peaks than CNG, with a 60% chance that any given cell will fire (Fig 3E).

We wanted to break down each Ca^2+^ transient into quantifiable components that could be compared across different conditions (Fig 3F). We defined a transient as a change in fluorescence that exceeded the standard deviation of the variability in the baseline by a factor of 26 [37]. For each fluorescent peak, we quantified the total cumulative peaks and the total number of peaks per ROI (S4–S10 Figs). The normalized △F of the peak (amplitude), the full width of the peak at half maximum (FWHM) and the inter-peak interval (S4–S10 Figs). Also, we examined the total number of Ca^2+^ transients recorded per experiment and recorded the time it took for a Ca^2+^ transient to occur post blue light stimulation.

### Voltage-gated sodium channel NavD creates faster Ca^2+^ transients

The bacterial sodium channel, NavRosDg217a (NavD) is characterized by a rapid activation state that depends on the cell depolarizing to -30mV, and a slow inactivation state (hundreds of milliseconds) that starts during the depolarization [34]. Co-expressing the NavD channel with Kir2.1 can potentially create a faster excitable system because the Kir2.1 hyperpolarizes the cell after each spontaneous depolarization caused by the sodium channel. We chose NavD because the mutant is slower than the mammalian sodium channels and in theory, we could create a system with depolarizations such that membrane depolarization events could be captured with slower imaging speeds (10Hz). A recent paper by Chen et al. found that varying the levels of Kir2.1, and Cav1.3 or Nav1.5 affected the membrane oscillations as recorded by electrophysiology [32]. We hypothesized that different levels of the Kir2.1 and NavD would lead to different corresponding Ca^2+^ responses.

HEK293 cells have endogenous connexin proteins, primarily connexin-45, that create gap junctions that electrically couple the cells [38, 39]. However, Kirkton and Bursac [20] found that when action potentials occur in a confluent layer of HEK293 cells expressing Kir2.1 and Nav1.5 they do not uniformly spread through the monolayer. Additional Connexin-43 produced rapid and robust action potentials. This is consistent with several other studies that have shown that including the Cx-43 protein increases the electrical gap junction coupling of HEK293 cells [20, 34, 40].

**Fig 4:**
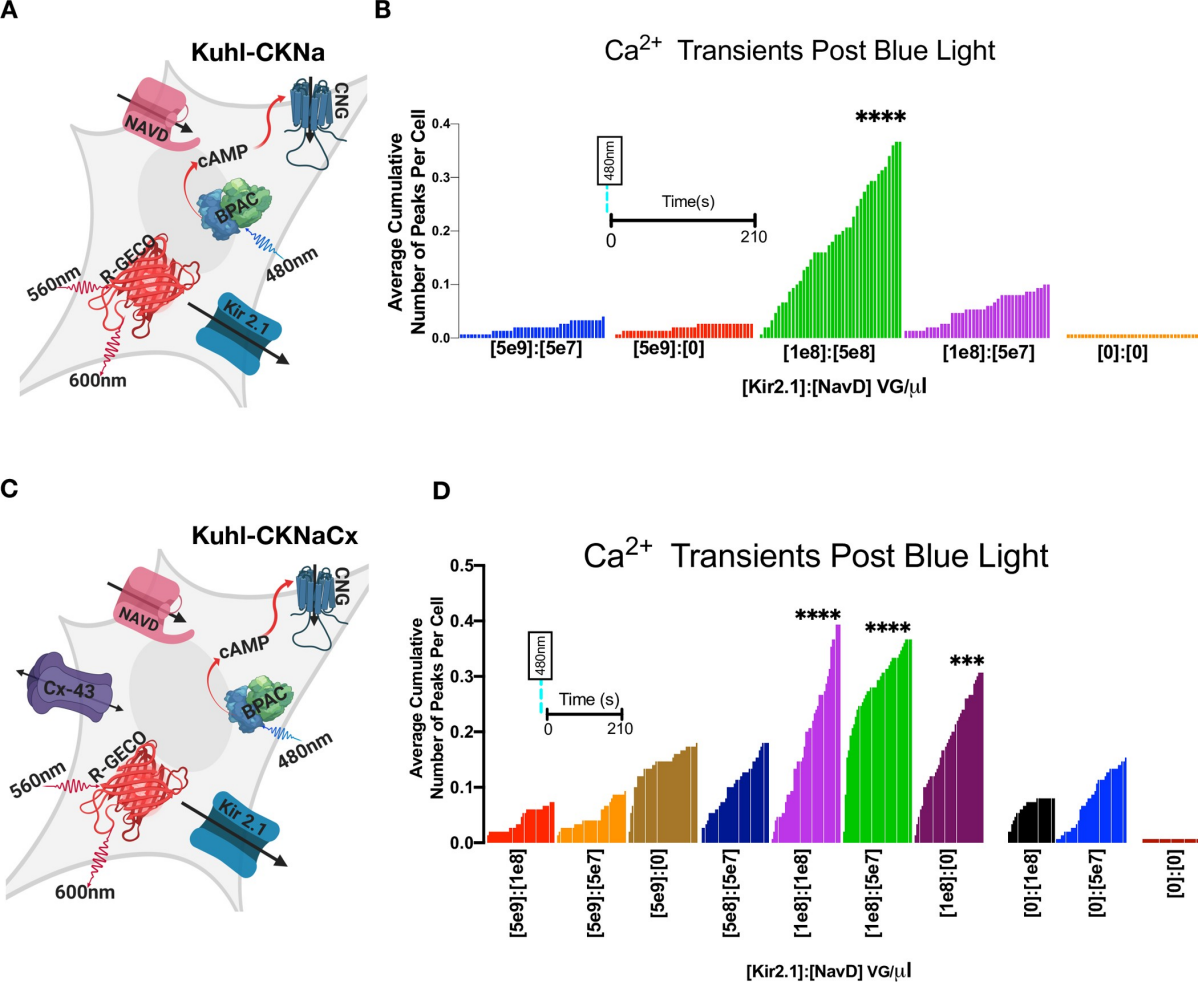
The Kir2.1 and NavD expression levels were optimized with and without Connexin-43. A) Cartoon depicting all the components expressed in the experimental Kuhl-CKNa cell (S1 Table). B) Shown is the average cumulative fluorescence peaks per cell across 210s for each indicated viral concentration of Kir2.1 and NavD (*n* = 150 cells per condition). The duration of imaging post blue light stimulus is the same for each condition (colored bar) in the graph. Differences were analyzed using Dunnett’s multiple comparisons test on the Ca^2+^ transient frequency (S4D Fig). (Kir2.1 1e8):(NavD 5e8) VG/*μ*L was significantly greater (****p ≤ .0001) than the control (Kir2.1 0):(NavD 0) VG/*μ*l. C) Cartoon depicting all the components transduced into the experimental Kuhl-CKNaCx cell. D) Shown is the average cumulative fluorescence peaks per cell across 210s for each indicated viral concentration of Kir2.1 and NavD (*n* = 150 cells per condition). Differences were analyzed using Dunnett’s multiple comparison test on the Ca^2+^ transient frequency (S5D Fig) compared to the control (Kir2.1 0):(NavD 0) VG/*μ*l. The experimental trials that were statistically greater were marked (****p ≤ .0001) and (***p ≤ .0002).

To test this we systematically transduced cells with varying ratios of Kir2.1 and NavD with and without Cx-43 while holding the viral concentrations of bPAC, CNG and R-GECO1 constant. The Kuhl-CKNa cells were transduced (S1 Table) and ~48 hours later the cells were stimulated with 20ms of blue light and images were collected with 561nm excitation. Upon activation of bPAC there were varying responses in each condition that are quantified (S4 and S5 Figs). Interestingly there seems to be a significant effect on the responses in the Kuhl-CKNaCx cells (Fig 4D) depending on the amount of (Kir2.1):(NavD) VG/*μ*L applied.

To determine the optimum (Kir2.1):(NavD) ratio we examined the average cumulative chance that a cell will fire by analyzing the total number of peaks for a well/total number of points collected from Inter-Peak (Fig 4B and 4D). Using the data from the Kuhl-CKNa cells we used the total number of peaks per ROI (S4D Fig) and ran a Dunnett's test (S2 Table) to examine if there was a statistical significance between the conditions. (Kir2.1 1e8):(NavD 5e8) VG/*μ*L had a significant increase (****p ≤ .0001) in the average cumulative number of peaks per cell (Fig 4B). In the Kuhl-CKNa system, there is a 35% chance that a cell will produce a significant Ca^2+^ peak and out of the cells that were suprathreshold there is a 43% chance of a second peak within 66s of the initial fluorescence peak (S4F Fig).

While there appears to be a range for optimal concentration using comparable amounts of the two channels in Kuhl-CKNaCx cells (Kir2.1 1e8) and (NavD 1e8) VG/uL evokes a significant response because there is a 40% chance that out of 150 cells a significant peak will occur in any given cell in 210s. Of the cells that were suprathreshold (S5D Fig) there is a 47% chance of a second peak within ~50s (S5F Fig) after the initial peak. Using the data from the total number of peaks per ROI (S5D Fig) we ran Dunnett's test (S2 Table) to examine if there was a statistical significance between the conditions. The experimental trials that were statistically greater than the control were marked (****p ≤ .0001, ***p ≤ .0002) on the graph (Fig 4D).

Introducing the NavD and Kir2.1 channel creates faster oscillating Ca^2+^ transients (S3 Fig) in the cell while introducing the Cx-43 increases coordinated activity between cells (S5 Fig). The differences in the mean duration that Ca^2+^ stayed elevated (S3 Fig) in the Kuhl-CKNa system with and without NavD and Cx-43 are shown. While both Kuhl cells containing CNG or HCN2 have random activity, there is a brief somewhat coordinated activity after a blue light stimulus (S7C and S8C Figs). The behavior of HCN2 was visually different from CNG. CNG has a Ca^2+^ bursting rosette pattern following a stimulus, while HCN2 seems to have a spreading wave-like pattern (S2 and S3 Movies).

### Repeatability

**Fig 5:**
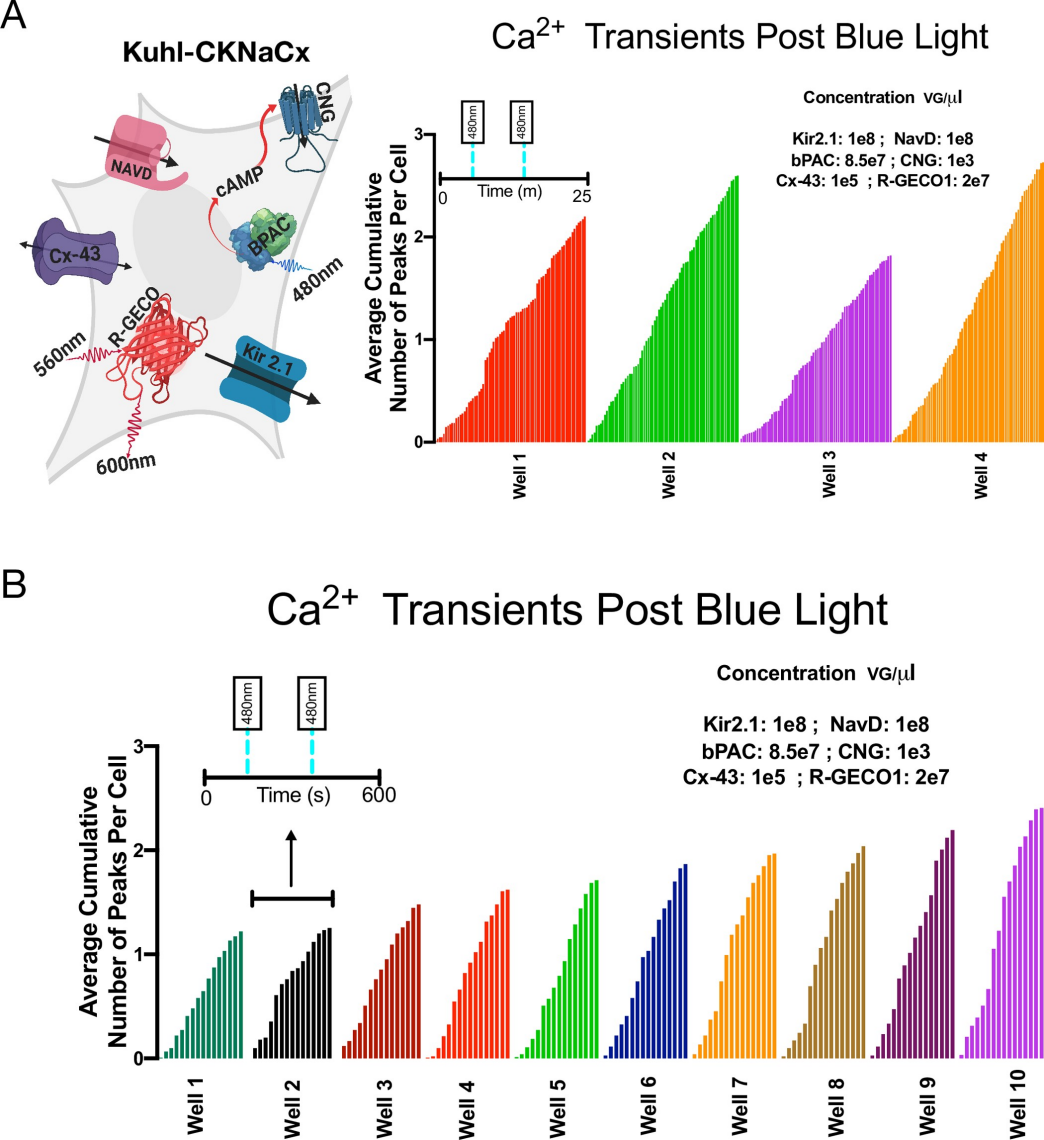
Kuhl-CKNaCx cells—consistent well to well activity. To be useful in a medium throughput screen, there needs to be consistent well to well activity, ensuring no false negatives. A) The representative cartoon depicts all the components transduced into the Kuhl-CKNaCx cell, and the average cumulative number of peaks per cell over four trials in 25m. B) The average cumulative number of peaks per cell over 10 trials in 15m. Tukey's multiple comparison test was run on the Kuhl-CKNaCx trials and found no significant difference between the wells.

Several iterative rounds of optimization were used to identify the right expression levels of each component to increase activity in terms of the total Ca^2+^ transients in every cell, and the total number of cells within the field of view having a significant Ca^2+^ peak (S4–S11 Figs). The optimized Kuhl-KCNaCx cells show remarkable, consistent activity when stimulated with blue light. In theory these Kuhl cells could be used to screen prototype activity sensors, new GECI and GEVI biosensors. Such a screen, however, would depend critically on consistent well to well activity. To measure how consistent the activity is from well to well, we integrated the activity seen in 14 different wells of cells. The representative cartoon (Fig 5A) depicts all the components transduced into the Kuhl-KCNaCx cell (S1 Table). One hundred percent of the 600 cells in the field of view will produce a significant peak. Fifty percent of the cells are likely to produce a second significant peak within ~94s ±32.

Another 10 wells (Fig 5B) were imaged for 15 minutes and stimulated with 20ms of blue light at 198s and 400s (S4 Movie). The cells have an oscillating firing pattern (S10C Fig) of 1 cycle every 20 seconds (S10F Fig) for each well. The well to well activity when measuring the average cumulative number of peaks per cell is fairly consistent with a mean of 1.8±.4. There is a 100% chance that 1,500 cells will produce a significant peak within 15 minutes (S10F Fig). Thirty percent are likely to produce a second significant peak within ~148s±89. Tukey's multiple comparison test did not find a significant difference between the average cumulative number of peaks per cell for the 14 trial wells with Kuhl-CKNaCx.

### Comparing green and red sensors in the same cell

While slow and laborious, an advantage of patch clamp fluorometry is that whole-cell voltage changes can be directly compared with fluorescence changes [41]. The all-optical Kuhl cell approach does not have the advantage of such an independent measurement. However, we reasoned that different colored sensors could be co-expressed and simultaneously imaged to arrive at a comparative measure. For example, a green sensor of known properties could be compared to a new one with red fluorescence. To test the feasibility of this approach, we used an optical splitter to compare the responses of R-GECO1 and G-GECO1 within the same cell.

**Fig 6:**
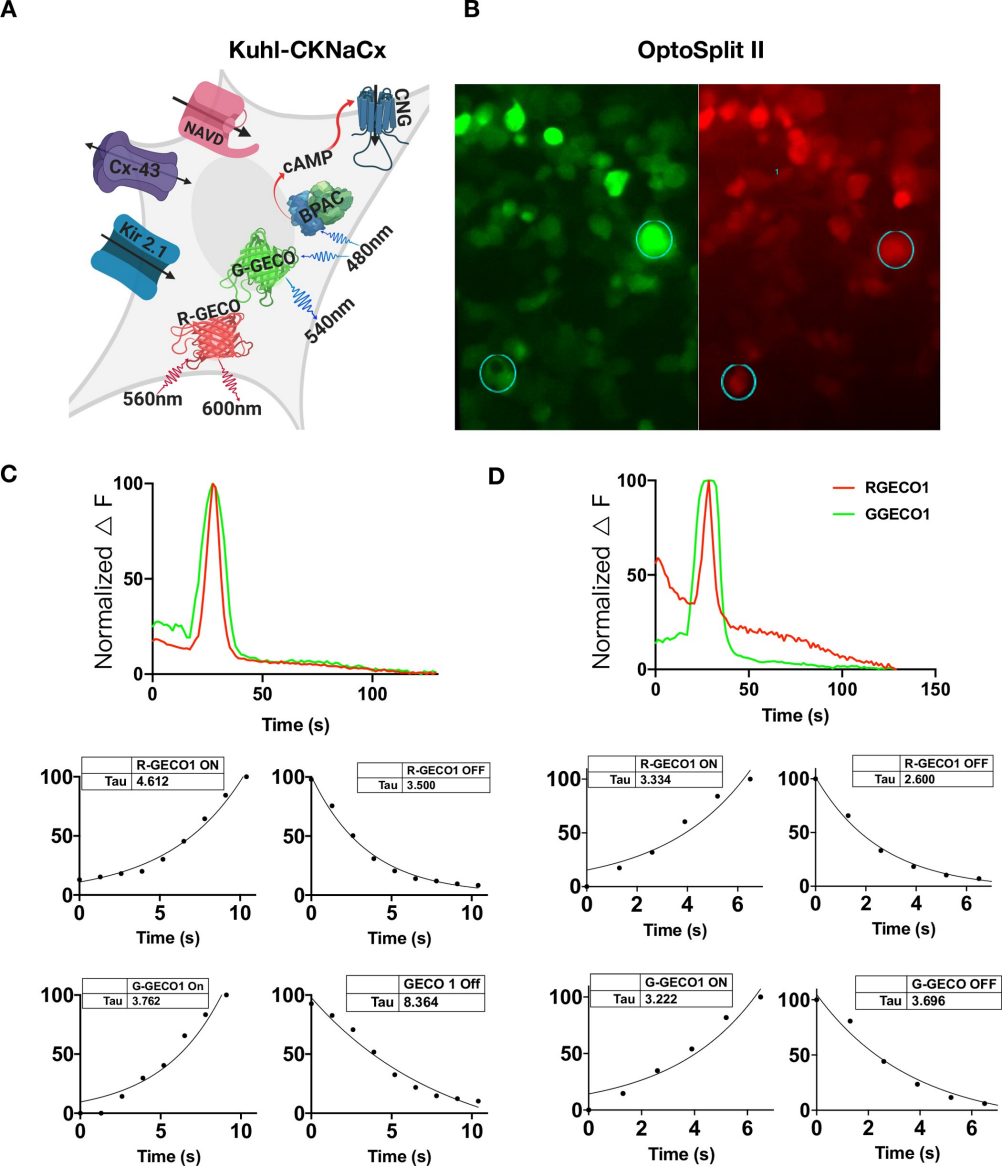
Red and green Ca^2+^ sensors can be directly compared in the same Kuhl cells using an OptoSplit II. A) Cartoon depicting all the components transduced into the Kuhl-CKNaCx cell. bPAC was transfected at a low level so that the cells could be imaged with green light continuously. B) The cells were simultaneously imaged with green and red wavelengths for 150s. The image is of the same cells in two different wavelengths and ROIs from both the G-GECO1 and R-GECO1 were obtained (circles and numbers). C) Representative traces from the ROI shown in the bottom left of panels 6B are of G-GECO1 and R-GECO1 fluorescence and their respective responses can be compared and plotted over time. The kinetics of both the red and green sensor from the same ROI is measured and plotted below the fluorescence trace. D) Representative traces from the upper right ROI in panels 6B. Both the R-GECO1 and G-GECO1 fluorescence and their respective responses in the same cell can be compared and plotted over time. The Tau_on/off_ of both the red and green sensor from the same ROI is measured and plotted below the fluorescence trace. The difference between G-GECO1 and R-GECO1 can be seen because it has a ~2 fold increase in the speed of its kinetics (K_d_).

The Kuhl-KCNaCx cells were transduced and 48 hours following the cells were imaged. In Fig 6 an image created by the OptoSplit II is shown where the green and red images of both sensors are simultaneously collected on two different portions of the EMCCD camera (S5 Movie). The fluorescence response from both sensors is plotted in (Fig 6C and 6D). This simple analysis shows that differences can be easily measured (Fig 6C and 6D) where the Tau_on/off_ of both signals was measured. R-GECO1 Tau_on_ was consistently slower than G-GECO1 and R-GECO1 Tau_off_ was consistently faster than G-GECO1 Tau_off_. Previous photophysical characterization of G-GECO1 and R-GECO1 show that there are relatively large differences between the two sensors. G-GECO1 has a faster K_on_ and R-GECO1 has a faster K_off_ [7]. G-GECO1 has a greater K_d, kinetic_ with a difference of 347 nM^1^.

### Green voltage sensors can be screened in Kuhl-CKNaCx cells

ArcLight is a downward going voltage sensor, meaning that it gets brighter when the membrane is hyperpolarized, and dimmer when it is depolarized [42]. It is pH sensitive and has been demonstrated to work in single-cell fly neurons, but requires high-intensity illumination to achieve an adequate signal-to-noise ratio [43].

**Fig 7:**
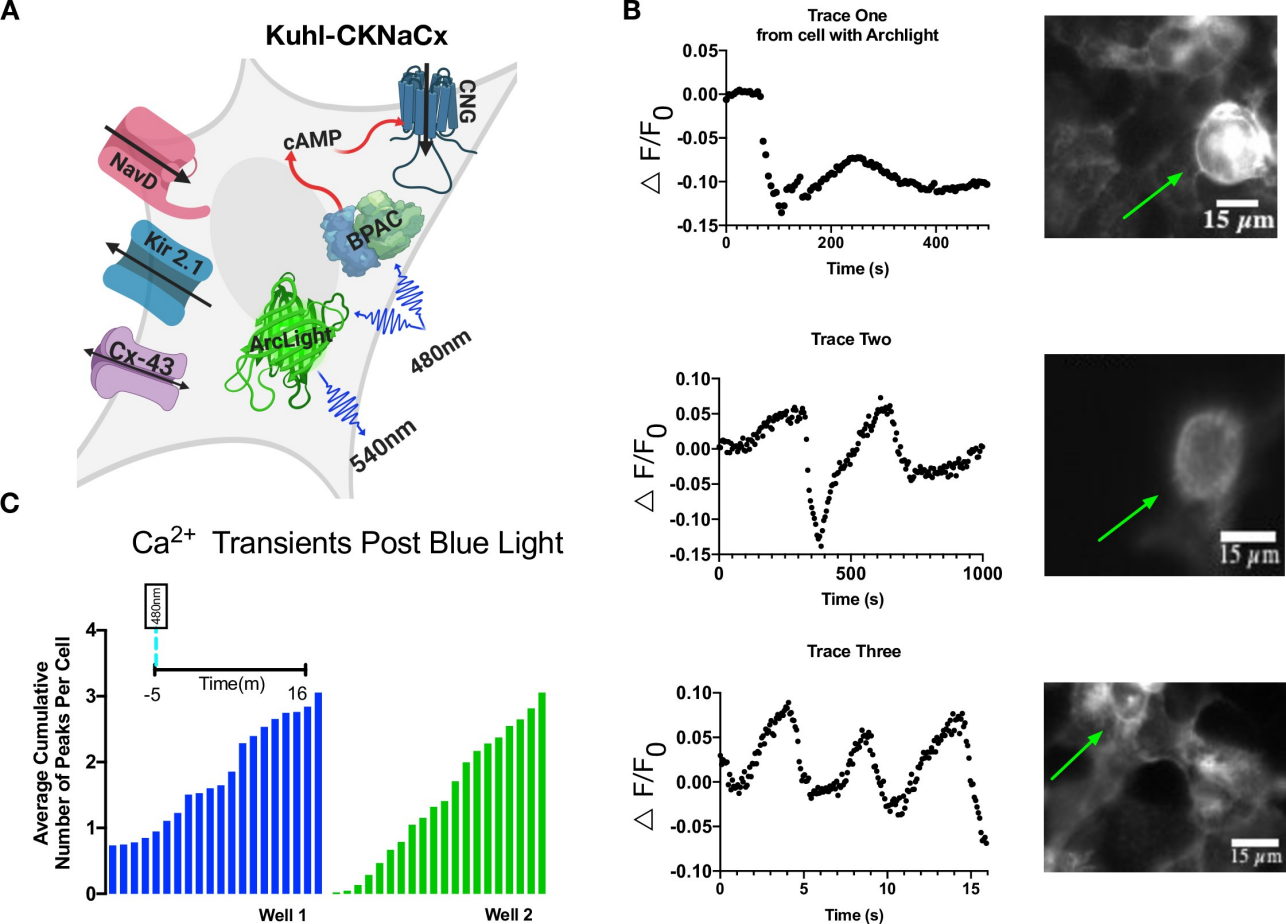
Voltage changes are visible with ArcLight in Kuhl-CKNaCx cells. A) Cartoon depicting all the components transduced into the Kuhl-CKNaCx cell. bPAC was stimulated continuously while exciting Arclight. B) Blue light activation of bPAC caused a slow depolarization that occurred over a few seconds. Representative traces from the ROIs show that voltage changes are visible using ArcLight. Images of ArcLight fluorescence response over time. The green arrows annotate which cells were used for the fluorescence traces. C) Total cumulative voltage fluorescence peaks from two separate wells that were imaged over 16 minutes are shown, both with an average of 3 fluorescence peaks per cell.

Slow depolarizations can be seen in the Kuhl-CKNaCx cell system (Fig 7) with a reasonable SNR. Comparing baseline fluorescence variation (standard deviation) with the amplitude of the fluorescence change reveals from 15 different cells an SNR of 5.5. The Kuhl-CKNaCx system shows promise for screening voltage sensor prototypes (S6–S8 Movies).

## Discussion

This Kuhl cell system was designed to be customizable and there are several important considerations to take into account when customizing for a particular activity sensor. Multiple channels could be exchanged for the ones used in this study. For example, a faster voltage-gated sodium channel would most likely create a faster action potential in the cells.

The Kuhl cells can potentially be used in medium throughput screens for better activity biosensors. The throughput of any screen, however, depends on the time budget necessary to scan a 96 or 384 well plate. In this study we arbitrarily defined activity as fluorescence peaks that exceeded the SNR of 26. That is, the peak was 26 times larger than the standard deviation of the baseline noise. Using this threshold, we found that out of 150 cells, imaging for 80s±30 was sufficient to detect 100 cells that would produce a Ca^2+^ peak above threshold. Recording the activity in a well for 80s would eliminate false negatives. The imaging time, plus the plate translation time, should result in a time budget of roughly 100 minutes per 96 well plate. While 400 to 500 wells a day is medium throughput, it is considerably better than the manual patch clamp fluorometry methods in use today.

We have shown that screening both red and green GECIs simultaneously in the Kuhl-CKNaCx system is possible, and that one sensor can be used to benchmark the other. Individual cells' dual GECI response can be measured for its brightness, kinetics (Tau), SNR, and sensitivity.

The improvement of the genetically encoded voltage indicators—GEVIs—has been slow due to limitations in the throughput involved in manual, whole-cell patch clamp fluorometry [13, 18]. In principal, the system described here will enable investigators to screen without field stimulation or any manual intervention. Voltage changes along the membrane of the HEK293 cells are visible and can be tracked in the Kuhl-CKNaCx cells using the GEVI ArcLight, since the action potential in these cells is quite slow, lasting seconds ~4s. We suggest that because we were able to benchmark Ca^2+^ indicators simultaneously that this should also work for voltage sensors. The prototype sensors could be expressed in Kuhl cells that also express a benchmark sensor such as GCAMP7f or Voltron [1, 44].

## Methods

### Growth conditions for HEK293 cells

A line of HEK293 were cultured in DMEM, 10% FBS, and 1% Penicillin (100 U/mL). For experiments, HE293 cells were plated in 96 well plastic plates at 100ml per well (~3–5 x10^4^ cells/mL).

### Plasmids

The HCN2 plasmid was kindly donated by Joan Lemire at Tufts University from Dr. Michael Levin’s lab. The NavRosDg217a was reverse translated using a human codon preference and synthesized as a gBblock by IDT (Coralville, Iowa). The cyclic nucleotide-gated olfactory channel (from *Rattus norvegicus*) has the following mutations, δ61–90/C460W/E583M and was synthesized as a gBblock by IDT [45]. pGEMTEZ-Kir2.1 was a gift from Richard Axel, Joseph Gogos & Ron Yu (Addgene plasmid #32641).

### Baculovirus

The channels Kir2.1, CNG, HCN2 and NavRosDg217a were packaged in BacMam (Montana Molecular, Bozeman, MT). The virus titers were: Kir2.1 3.6x10^10^ VG/mL, bPAC 8.59 x 10^10^ VG/mL, CNG 3.55x10^10^ VG/mL, and NavRosDg217a 7.26x 10^10^ VG/mL. The following bio-sensors in baculovirus were obtained from Montana Molecular: R-cADDis (titer 5.06x10^10 VG/mL); R-GECO (2x10^10^ VG/mL); G-GECO (9.17x10^10^ VG/mL); and Arclight (2x10^10^ VG/mL). S1 Table depicts each experimental condition and the final viral concentrations used.

### Transduction

HEK-293 cells were prepared and transduced following the manufacturer's recommended protocol. Briefly, HEK293 cells were plated in 96 well plastic plates (48 x10^3^ cells/well) and transduced with the appropriate mix of viruses and the HDAC inhibitor sodium butyrate (2mM final concentration). Two days later, the media was exchanged with PBS, tyrodes buffer, or Fluorobrite. The best results were consistently obtained with PBS.

### An ASI modular microscope

The experiments (Figs 1–5 and 7) were conducted using wide field imaging on an ASI-XYZ stage fitted with a modular infinity microscope. The objective imaged onto the detector chip of a Hamamatsu ORCA-Flash 4.0 scientific-CMOS camera. The images were collected using either MATLAB scripts that controlled the camera, ASI stage, SH1-Thorlabs shutter, and ThorLabs DC4100 Four Channel-LED Driver, or by using μManager [46]. A custom illumination system was arranged. Briefly, a dichroic mirror was positioned at the entrance of the microscope to combine blue light LED illumination with 561 laser illumination. An SH1-Thorlabs shutter was used to create brief illumination from a blue ThorLABS LED (488nm) for rapid stimulation of bPAC. At 100% the blue light illumination was 70mW/cm^2^. The yellow illumination was provided with a Sapphire laser (561nm, 50mW, Coherent). The laser beam was steered with two mirrors (arranged in a periscope) into an entrance aperture of the ASI microscope. Before entering the microscope, the laser beam passed through a 50^0^ diffuser (ED1 C50 MD, Thorlabs) placed in the focus of an f = 20 mm aspheric lens (ACL2520U, Thorlabs) that collimated the beam for further traveling into the microscope.

#### Optosplitter

To simultaneously collect images of both G-GECO and R-GECO, we used an optical splitter (CAIRN optosplit II, Faversham Kent ME13 8UP). This makes it possible to simultaneously collect both the green and red images on a single camera. An Olympus IX81 was fitted with LED illumination (470 nM, 565 nM, ThorLabs) where the power to the LEDs can be independently adjusted. A Pinkel filter set was used to simultaneously image both G-GECO and R-GECO (GFP/DsRed-2X-A-000, Semrock, Rochester, New York). The CAIRN splitter was positioned in between the microscope and EMCCD camera such that a dichroic filter (561 nM, Semrock, Rochester, New York) split the green and red emission into two optical paths. The red emission passed through an additional band pass filter (617/70, Semrock, Rochester, New York) to eliminate any potential green emission from the G-GECO.

#### Analysis

Image data was stored in a Z-stack tiff file and loaded into the FIJI distribution of the ImageJ software [47]. The initial frame of the stack was eliminated from all analysis to correct for photoactivation of R-GECO1 upon bluelight stimulation of bPAC [48]. The cells were selected using a freehand ROI surrounding the cell of interest. The average total intensity pixel value within the ROI for each frame was collected using the time series analyzer plugin and saved as a .csv file. The raw fluorescence trace data was loaded into MATLAB.

The analysis was done in MATLAB using the Find Peaks function in the Signal Processing Toolbox. The raw fluorescence traces were rescaled from zero to 10,000 and normalized to zero. The data was smoothed using ‘sgolay’ function. The main peak prominence threshold was set to 50.5 with an SNR of 26±10. The Signal-to-noise ratio was defined as the average of the peaks amplitudes that were defined using the peak finder thresholder by the standard deviation (S.D.) of the signal before blue light stimulus. We examined the SNR for Kuhl-CKNaCx cells over 5 individual trials which were averaged over 15 trials. The FindPeaks function returns a vector with the local maxima (peaks) from the ROI trace data. The Peak Finder in MATLAB marks each fluorescence peak by its location in time and records the number of peaks per ROI. In addition, it returns the widths of the peak and the prominence of the peak. The time that a fluorescence peak occurred during imaging was recorded. The inter-event interval is the absolute difference between each consecutive peak. The FWHM and inter-event interval time was adjusted for exposure times. The total cumulative number of peaks for an entire trial was measured. Each fluorescence peak was measured from zero to the maximum peak intensity (normalized F) using the MATLAB Peak Finder. The time and total quantity of a fluorescence peak in a ROI were recorded. To determine the chance that a cell will fire twice was determined by (total number of peaks for a well/total number of points collected from Inter-Peak interval).

## Supporting information

S1 Fig Fluorescence trace of cells with Kir2.1, CNG, R-GECO1 (Kuhl-CK). Without bPAC blue light stimulus is ineffective at creating a response within cells.
(TIF)

S2 Fig Representative traces. Raw fluorescence traces from Kuhl-CK AND Kuhl-HK. 20s of blue light stimulus leads to varied fluctuating responses in each cell.
(TIF)

S3 Fig Introduction of NavD to Kuhl and Cx-43 increases the speed of the Ca^2+^ transients. A) The most significant wavelengths from each optimized set was included in the data to show the increase in Ca^2+^ transient speed.
(TIF)

S4 Fig Features of the [Kir2.1]:[NavD] optimized Kuhl-CKNa cells and their Ca^2+^ transients. A) The total peaks for each Ca^2+^ transient within a trial was recorded, and the experiment with [1e8 Kir2.1]:[5e8 NavD] VG/*μ*L had the greatest recorded peaks. B) The △F is the difference between the baseline fluorescence and the maximum fluorescence of each peak. The mean and S.E.M of the Ca^2+^ transients in [Kir2.1]:[NavD] optimized Kuhl-CKNa cells C) Blue line indicates point of 20s blue light stimulus. The time of each fluorescence peak was recorded and is indicated by a black dot. D) The total number of Ca^2+^ transients per cell (ROI) for experimental [Kir2.1]:[NavD] optimized Kuhl-CKNa cells. E) Representative duration of elevated R-GECO1 fluorescence over time per Ca^2+^ transient. Black dot indicates the Ca^2+^ transients FWHM for each peak. F) Black dot indicates the time between Ca^2+^ transient events per cell. Red bars indicate mean and ± S.E.M (*n* = 150 cells per condition).
(TIF)

S5 Fig Features of the [Kir2.1]:[NavD] optimized Kuhl-CKNaCx cells and their Ca^2+^ transients. A) The total peaks for each Ca^2+^ transient within a trial was recorded, and the experiment with [1e8 Kir2.1]:[1e8 NavD] VG/*μ*L had the greatest recorded peaks. B) The △F is the difference between the baseline fluorescence and the maximum fluorescence of each peak. The mean and S.E.M of the Ca^2+^ transients in [Kir2.1]:[NavD] optimized Kuhl-CKNaCx cells C) Blue line indicates point of 20s blue light stimulus. The time of each fluorescence peak was recorded and is indicated by a black dot. D) The total number of Ca^2+^ transients per cell (ROI) for experimental [Kir2.1]:[NavD] optimized Kuhl-CKNaCx. E) Representative duration of elevated R-GECO1 fluorescence over time per Ca^2+^ transient. Black dot indicates the Ca^2+^ transients FWHM for each peak. F) Black dot indicates the time between Ca^2+^ transient events per cell. Red bars indicate mean and ± S.E.M (*n* = 150 cells per condition).
(TIF)

S6 Fig Features of the Cx-43 optimized Kuhl-CKNaCx cells and their Ca^2+^ transients. Corresponding S8 Fig. A) The total peaks for each Ca^2+^ transient within a trial was recorded, and the experiment with [Cx 1e5] VG/*μ*L had the greatest recorded peaks. B) The △F is the difference between the baseline fluorescence and the maximum fluorescence of each peak. The mean and S.E.M of the Ca^2+^ transients in Cx-43 optimized Kuhl-CKNaCx cells. C) Blue line indicates point of 20s blue light stimulus. The time of each fluorescence peak was recorded and is indicated by a black dot. D) The number of Ca^2+^ transients per cell (ROI) for Cx-43 optimized Kuhl-CKNaCx cells. E) Representative duration of elevated R-GECO1 fluorescence over time per Ca^2+^ transient. Black dot indicates the Ca^2+^ transients FWHM for each peak. F) Black dot indicates the time between Ca^2+^ transient events per cell. Red bars indicate mean and ± S.E.M (*n* = 150 cells per condition).
(TIF)

S7 Fig Features of the CNG optimized Kuhl-CKNaCx cells and their Ca^2+^ transients. Corresponding S9 Fig. A) The total peaks for each Ca^2+^ transient within a trial was recorded, and the experiment with [CNG 1e3] VG/μL had the greatest recorded peaks. B) The △F is the difference between the baseline fluorescence and the maximum fluorescence of each peak. The mean and S.E.M of the Ca^2+^ transients in CNG optimized Kuhl-CKNaCx cells C) Blue line indicates point of 20s blue light stimulus. The time of each fluorescence peak was recorded and is indicated by a black dot. The wells without CNG and Cx were very interesting because it appears that without blue light there is a D) The total number of Ca^2+^ transients per cell (ROI) for experimental CNG optimized Kuhl-CKNaCx cells. E) Representative duration of elevated R-GECO1 fluorescence over time per Ca^2+^ transient. Black dot indicates the Ca^2+^ transients FWHM for each peak. F) Black dot indicates the time between Ca^2+^ transient events per cell. Red bars indicate mean and ± S.E.M (*n* = 150 cells per condition).
(TIF)

S8 Fig Features of the HCN2 optimized Kuhl-HKNaCx cells and their Ca^2+^ transients. Corresponding S10 Fig. A) The total peaks for each Ca^2+^ transient within a trial (T = 300s) was recorded. The experiment with [HCN2 1e2] VG/μL had the greatest recorded peaks. B) The △F is the difference between the baseline fluorescence and the maximum fluorescence of each peak. The mean and S.E.M of the Ca^2+^ transients in HCN2 optimized Kuhl-HKNaCx cells C) Blue line indicates point of 20s blue light stimulus. The time of each fluorescence peak was recorded and is indicated by a black dot. D) The total number of Ca^2+^ transients per cell (ROI) for experimental HCN2 optimized Kuhl-HKNaCx. E) Representative duration of elevated R-GECO1 fluorescence over time per Ca^2+^ transient. Black dot indicates the Ca^2+^ transients FWHM for each peak. F) Black dot indicates the time between Ca^2+^ transient events per cell. Red bars indicate mean and ± S.E.M (*n* = 150 cells per condition).
(TIF)

S9 Fig Features of the optimized Kuhl-CKNaCx cells and their Ca^2+^ transients. Corresponding S11A Fig. A) The total peaks for each Ca^2+^ transient within a trial (T = 25m) was recorded. B) The △F is the difference between the baseline fluorescence and the maximum fluorescence of each peak. The mean and S.E.M of the Ca^2+^ transients in optimized Kuhl-CKNaCx cells C) Blue line indicates point of 20s blue light stimulus. The time of each fluorescence peak was recorded and is indicated by a black dot. D) The total number of Ca^2+^ transients per cell (ROI) for optimized Kuhl-CKNaCx cells. Representative duration of elevated R-GECO1 fluorescence over time per Ca^2+^ transient. Black dot indicates the Ca^2+^ transients FWHM for each peak. F) Black dot indicates the time between Ca^2+^ transient events per cell. Red bars indicate mean and ± S.E.M (*n* = 150 cells per condition).
(TIF)

S10 Fig Features of the optimized Kuhl-CKNaCx cells and their Ca^2+^ transients. Corresponding S11B Fig. A) The total peaks for each Ca^2+^ transient within a trial (T = 25m) was recorded. B) The △F is the difference between the baseline fluorescence and the maximum fluorescence of each peak. The mean and S.E.M of the Ca^2+^ transients in optimized Kuhl-CKNaCx cells C) Blue line indicates point of 20s blue light stimulus. The time of each fluorescence peak was recorded and is indicated by a black dot. D) The total number of Ca^2+^ transients per cell (ROI) for optimized Kuhl-CKNaCx. Representative duration of elevated R-GECO1 fluorescence over time per Ca^2+^ transient. Black dot indicates the Ca^2+^ transients FWHM for each peak. F) Black dot indicates the time between Ca^2+^ transient events per cell. Red bars indicate mean and ± S.E.M (*n* = 150 cells per condition).
(TIF)

S11 Fig Multiple rounds of optimization. A) Activity was optimized by varying the viral concentration of CNG. Differences were analyzed using Dunnett’s multiple comparison test on the Ca^2+^ transient frequency (S7D Fig) compared to the control (CNG 0) VG/μl. The experimental trials that were statistically greater than the control were marked (****p ≤ .0001) on the graph. B) The gap junctions between cells using connexin-43 was optimized by varying levels of viral titer. C) Activity was optimized by varying the viral concentration of HCN2. Shown are the average cumulative fluorescence peaks per cell across 360s for each indicated viral concentration of HCN2 (*n* = 150 cells per condition).
(TIF)

S1 Table Experimental setup for each figure. The table shows the experimental set up, the final viral concentration used, and their corresponding figures.
(TIF)

S2 Table Dunnett’s multiple comparisons test. The table shows all test run using Dunnetts where stated in the manuscript.
(TIF)

S1 Movie Bright subcellular flashes of Ca2+. The authors do not know what these come from.
(AVI)

S2 Movie The temporal/spatial movement of CNG in Kuhl-CKNaCx cells. Rose like patterns. The behavior of HCN2 was visually different from CNG. While both wells have random activity, there is a brief somewhat coordinated activity after a blue light stimulus (S7C and S8C Figs). S2 and S3 Movies show the movement of CNG and HCN2. CNG has a Ca^2+^ bursting rosette pattern following a stimulus, while HCN2 seems to have a spreading wave-like pattern.
(AVI)

S3 Movie The temporal/spatial movement of HCN2 in Kuhl-HKNaCx cells. Wave-like spreading pattern.
(AVI)

S4 Movie A well from the Kuhl-CKNaCx 10 well experiment. Cells imaged for 15 minutes with robust, repeatable activity.
(AVI)

S5 Movie G-GECO1 and R-GECO1 can be imaged simultaneously. An image created by the OptoSplit II is shown where the green and red emission from both sensors are split and imaged on two different portions of the EMCCD camera.
(AVI)

S6 Movie Whole well image of ArcLight activity. Activity of Kuhl-CKNaCx cells with the GEVI ArcLight.
(AVI)

S7 Movie Combined images of ArcLight activity. Activity of Kuhl-CKNaCx cells with the GEVI ArcLight from the same well.
(AVI)

S8 Movie Combined images of ArcLight activity. Activity of Kuhl-CKNaCx cells with the GEVI ArcLight from the same well.
(MOV)
