# Supplementary figures and images for: Optically activated, customizable, excitable cells
Source: PLoS One. 2020 Dec 30;15(12):e0229051. doi: 10.1371/journal.pone.0229051 (PMC7773186; doi:10.1371/journal.pone.0229051)

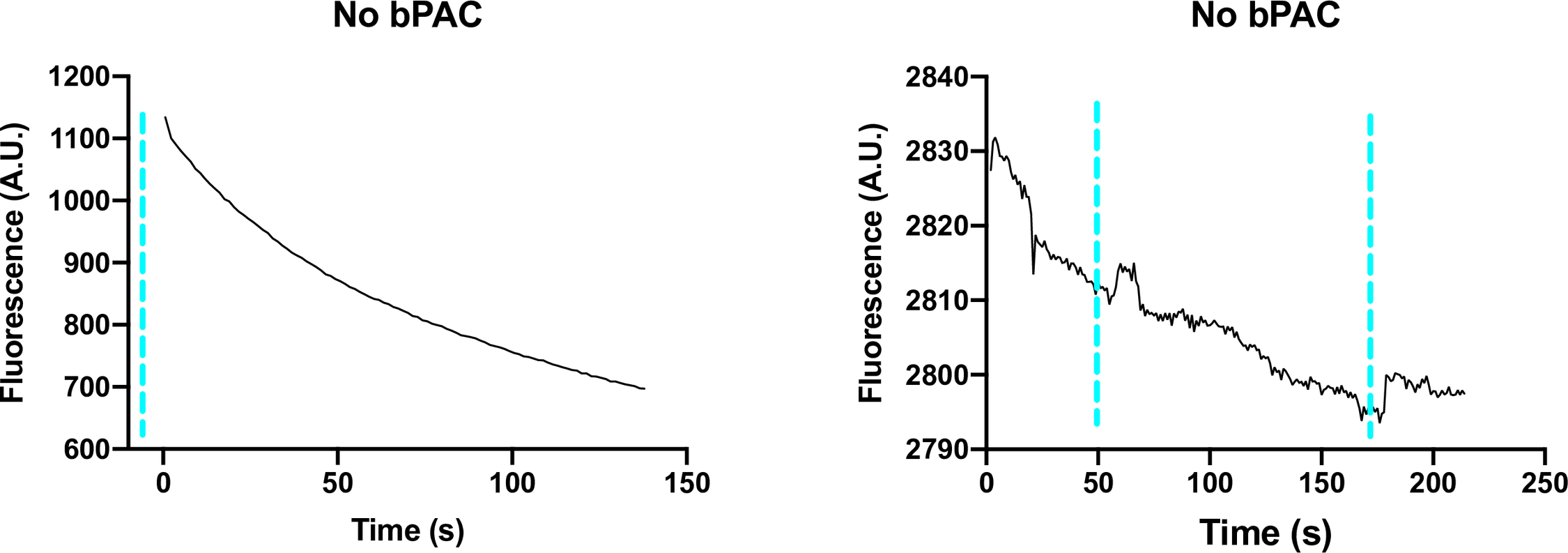

Supplement: S1 Fig — Without bPAC blue light stimulus is ineffective at creating a response within cells. (TIF) [file pone.0229051.s001.tif]

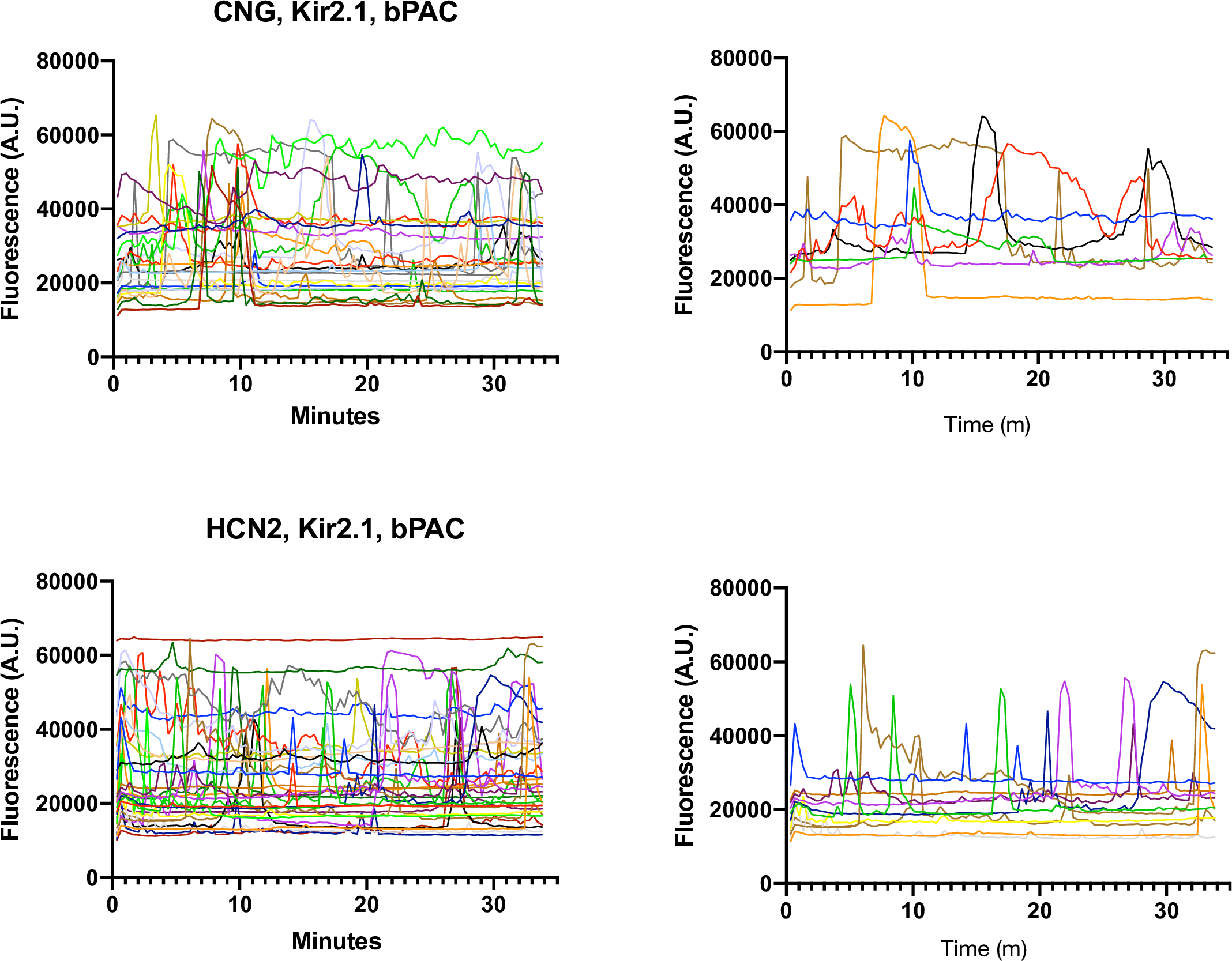

Supplement: S2 Fig — Raw fluorescence traces from Kuhl-CK AND Kuhl-HK. 20s of blue light stimulus leads to varied fluctuating responses in each cell. (TIF) [file pone.0229051.s002.tif]

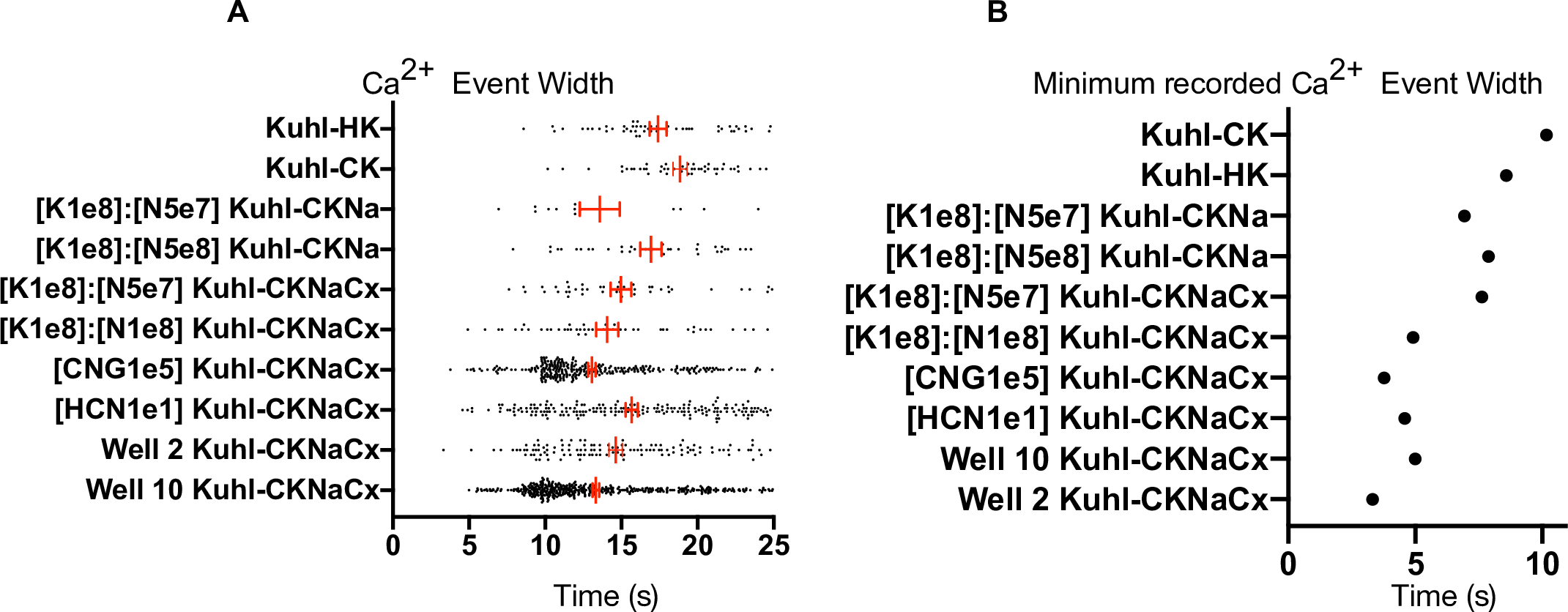

Supplement: S3 Fig — A) The most significant wavelengths from each optimized set was included in the data to show the increase in Ca2+ transient speed. (TIF) [file pone.0229051.s003.tif]

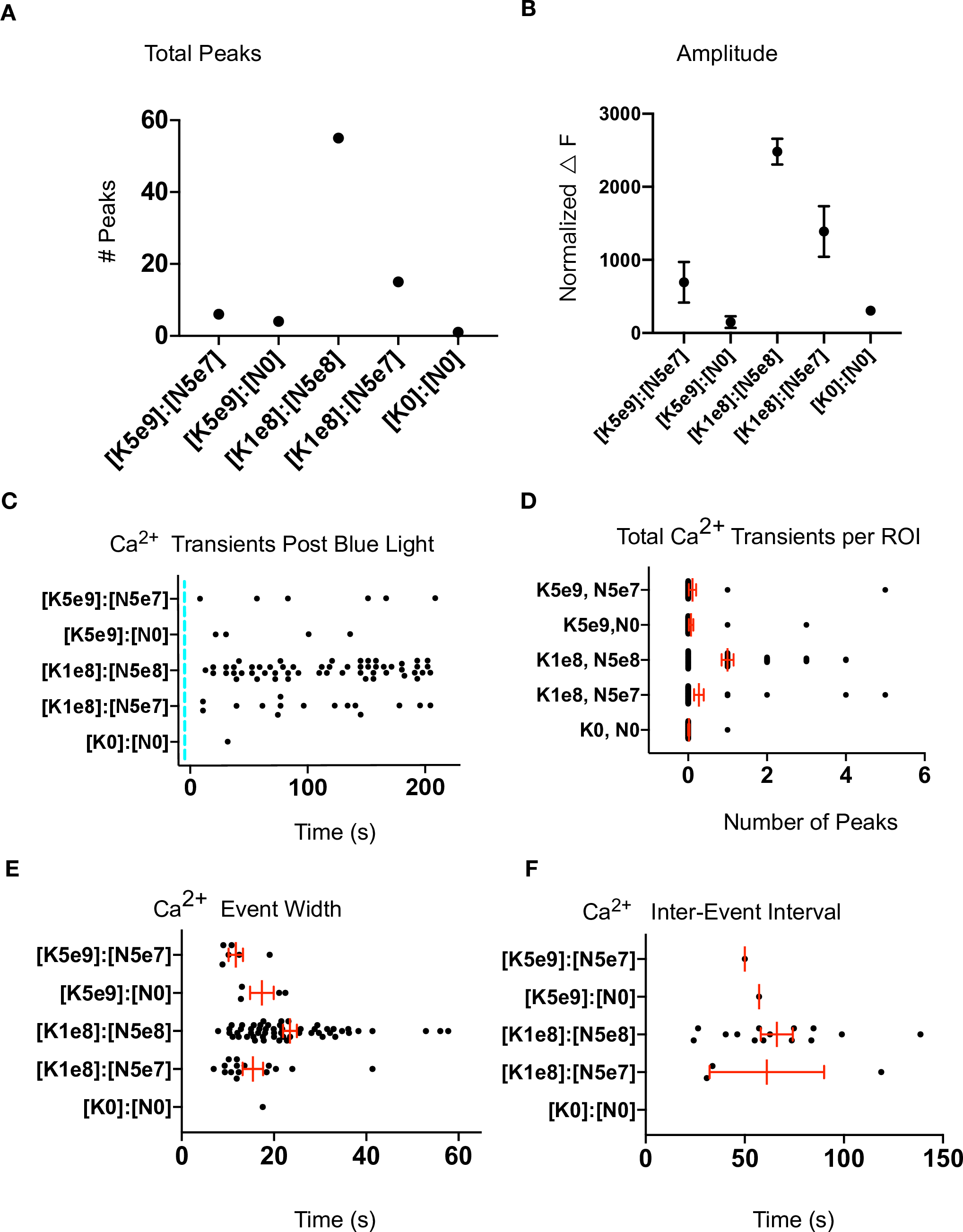

Supplement: S4 Fig — A) The total peaks for each Ca2+ transient within a trial was recorded, and the experiment with [1e8 Kir2.1]:[5e8 NavD] VG/μL had the greatest recorded peaks. B) The △F is the difference between the baseline fluorescence and the maximum fluorescence of each peak. The mean and S.E.M of the Ca2+ transients in [Kir2.1]:[NavD] optimized Kuhl-CKNa cells C) Blue line indicates point of 20s blue light stimulus. The time of each fluorescence peak was recorded and is indicated by a black dot. D) The total number of Ca2+ transients per cell (ROI) for experimental [Kir2.1]:[NavD] optimized Kuhl-CKNa cells. E) Representative duration of elevated R-GECO1 fluorescence over time per Ca2+ transient. Black dot indicates the Ca2+ transients FWHM for each peak. F) Black dot indicates the time between Ca2+ transient events per cell. Red bars indicate mean and ± S.E.M (n = 150 cells per condition). (TIF) [file pone.0229051.s004.tif]

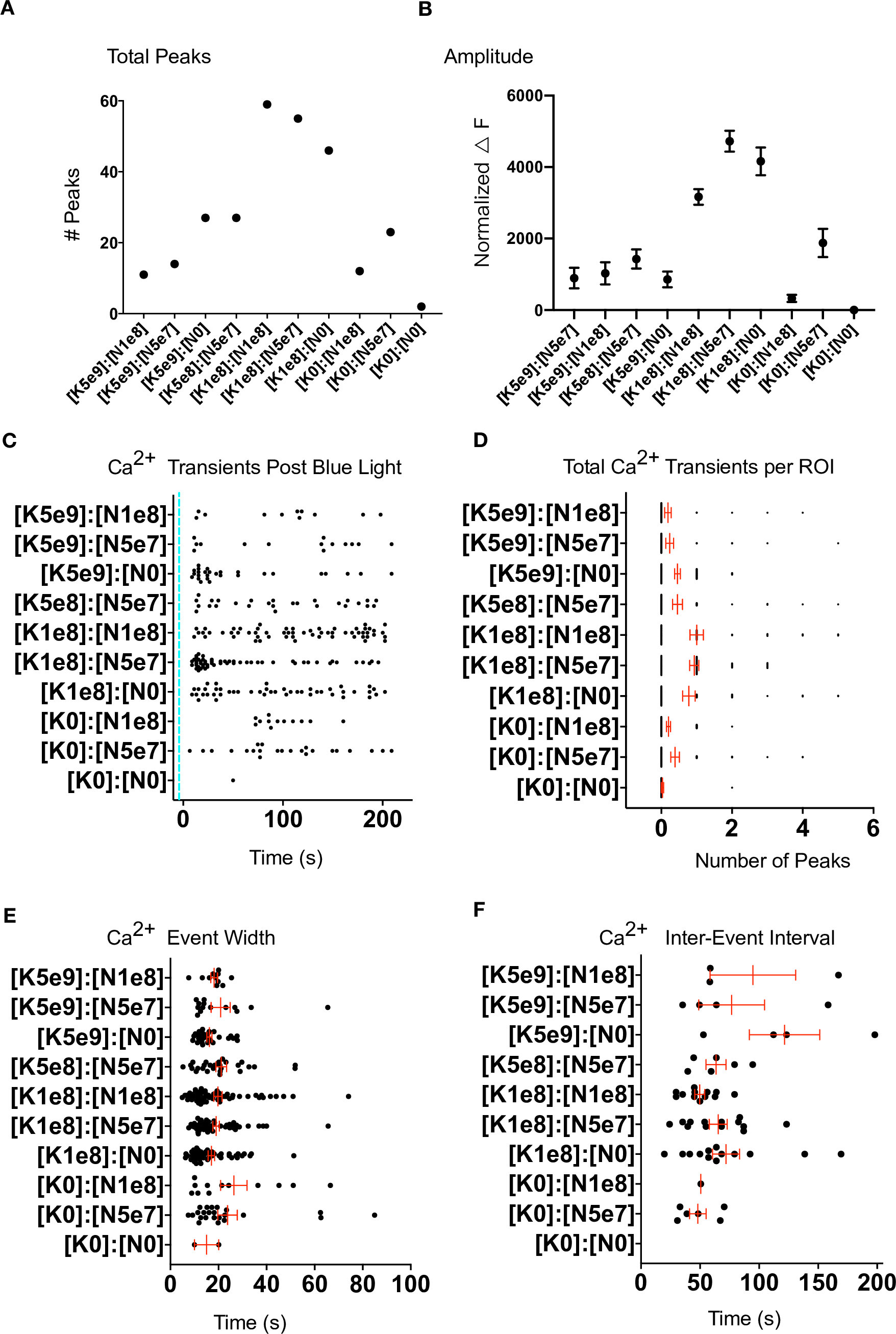

Supplement: S5 Fig — A) The total peaks for each Ca2+ transient within a trial was recorded, and the experiment with [1e8 Kir2.1]:[1e8 NavD] VG/μL had the greatest recorded peaks. B) The △F is the difference between the baseline fluorescence and the maximum fluorescence of each peak. The mean and S.E.M of the Ca2+ transients in [Kir2.1]:[NavD] optimized Kuhl-CKNaCx cells C) Blue line indicates point of 20s blue light stimulus. The time of each fluorescence peak was recorded and is indicated by a black dot. D) The total number of Ca2+ transients per cell (ROI) for experimental [Kir2.1]:[NavD] optimized Kuhl-CKNaCx. E) Representative duration of elevated R-GECO1 fluorescence over time per Ca2+ transient. Black dot indicates the Ca2+ transients FWHM for each peak. F) Black dot indicates the time between Ca2+ transient events per cell. Red bars indicate mean and ± S.E.M (n = 150 cells per condition). (TIF) [file pone.0229051.s005.tif]

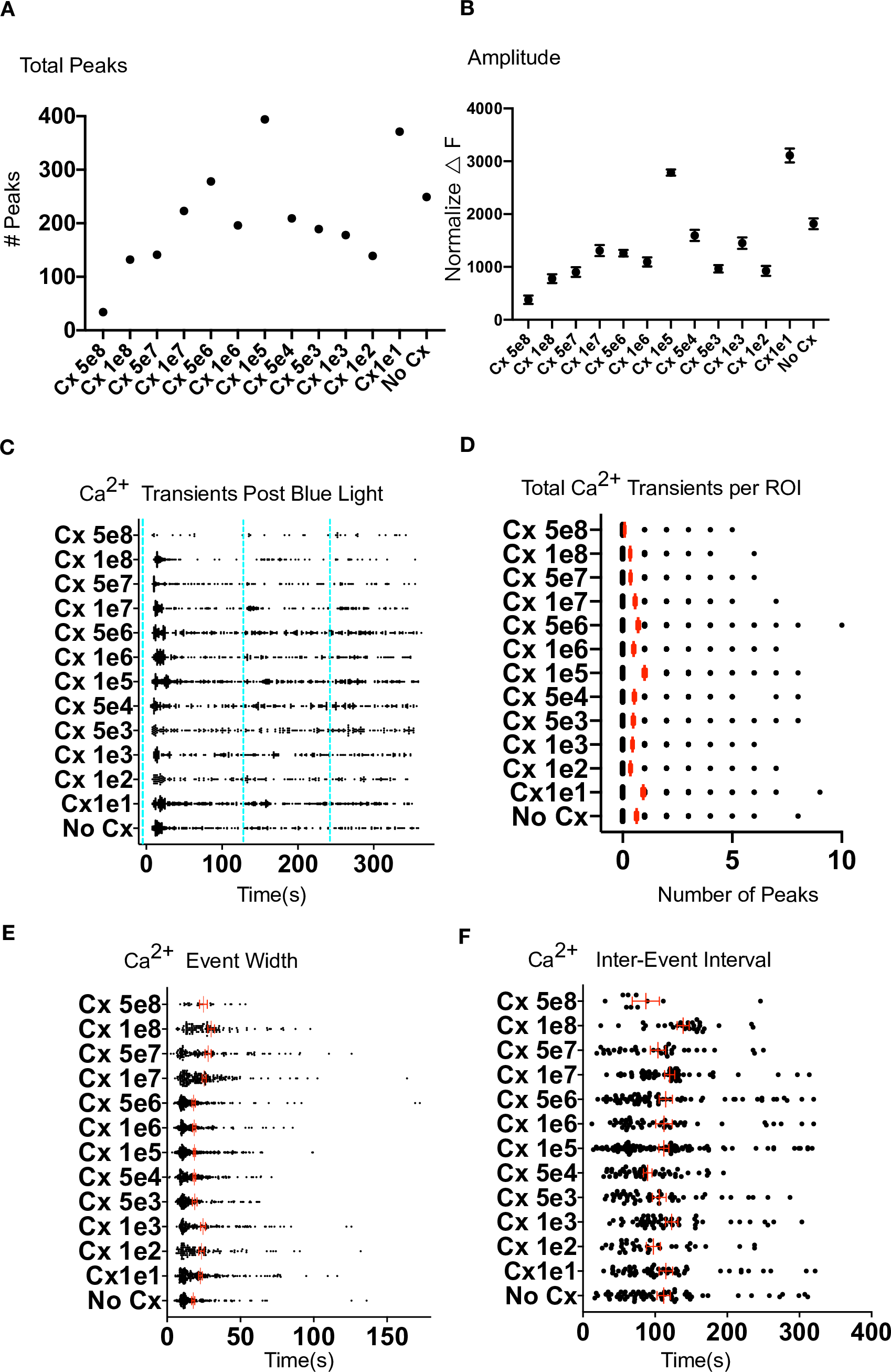

Supplement: S6 Fig — Corresponding S8 Fig. A) The total peaks for each Ca2+ transient within a trial was recorded, and the experiment with [Cx 1e5] VG/μL had the greatest recorded peaks. B) The △F is the difference between the baseline fluorescence and the maximum fluorescence of each peak. The mean and S.E.M of the Ca2+ transients in Cx-43 optimized Kuhl-CKNaCx cells. C) Blue line indicates point of 20s blue light stimulus. The time of each fluorescence peak was recorded and is indicated by a black dot. D) The number of Ca2+ transients per cell (ROI) for Cx-43 optimized Kuhl-CKNaCx cells. E) Representative duration of elevated R-GECO1 fluorescence over time per Ca2+ transient. Black dot indicates the Ca2+ transients FWHM for each peak. F) Black dot indicates the time between Ca2+ transient events per cell. Red bars indicate mean and ± S.E.M (n = 150 cells per condition). (TIF) [file pone.0229051.s006.tif]

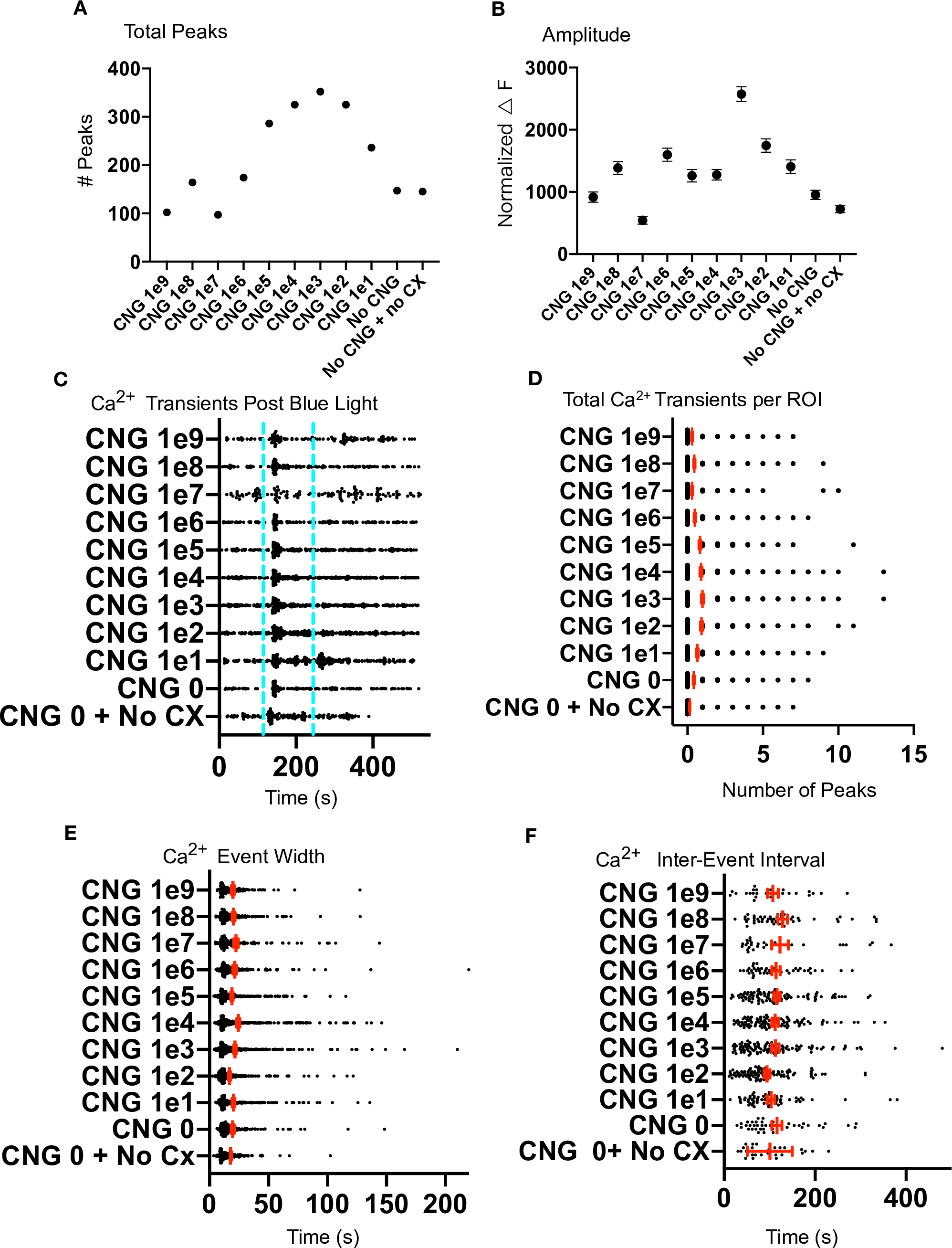

Supplement: S7 Fig — Corresponding S9 Fig. A) The total peaks for each Ca2+ transient within a trial was recorded, and the experiment with [CNG 1e3] VG/μL had the greatest recorded peaks. B) The △F is the difference between the baseline fluorescence and the maximum fluorescence of each peak. The mean and S.E.M of the Ca2+ transients in CNG optimized Kuhl-CKNaCx cells C) Blue line indicates point of 20s blue light stimulus. The time of each fluorescence peak was recorded and is indicated by a black dot. The wells without CNG and Cx were very interesting because it appears that without blue light there is a D) The total number of Ca2+ transients per cell (ROI) for experimental CNG optimized Kuhl-CKNaCx cells. E) Representative duration of elevated R-GECO1 fluorescence over time per Ca2+ transient. Black dot indicates the Ca2+ transients FWHM for each peak. F) Black dot indicates the time between Ca2+ transient events per cell. Red bars indicate mean and ± S.E.M (n = 150 cells per condition). (TIF) [file pone.0229051.s007.tif]

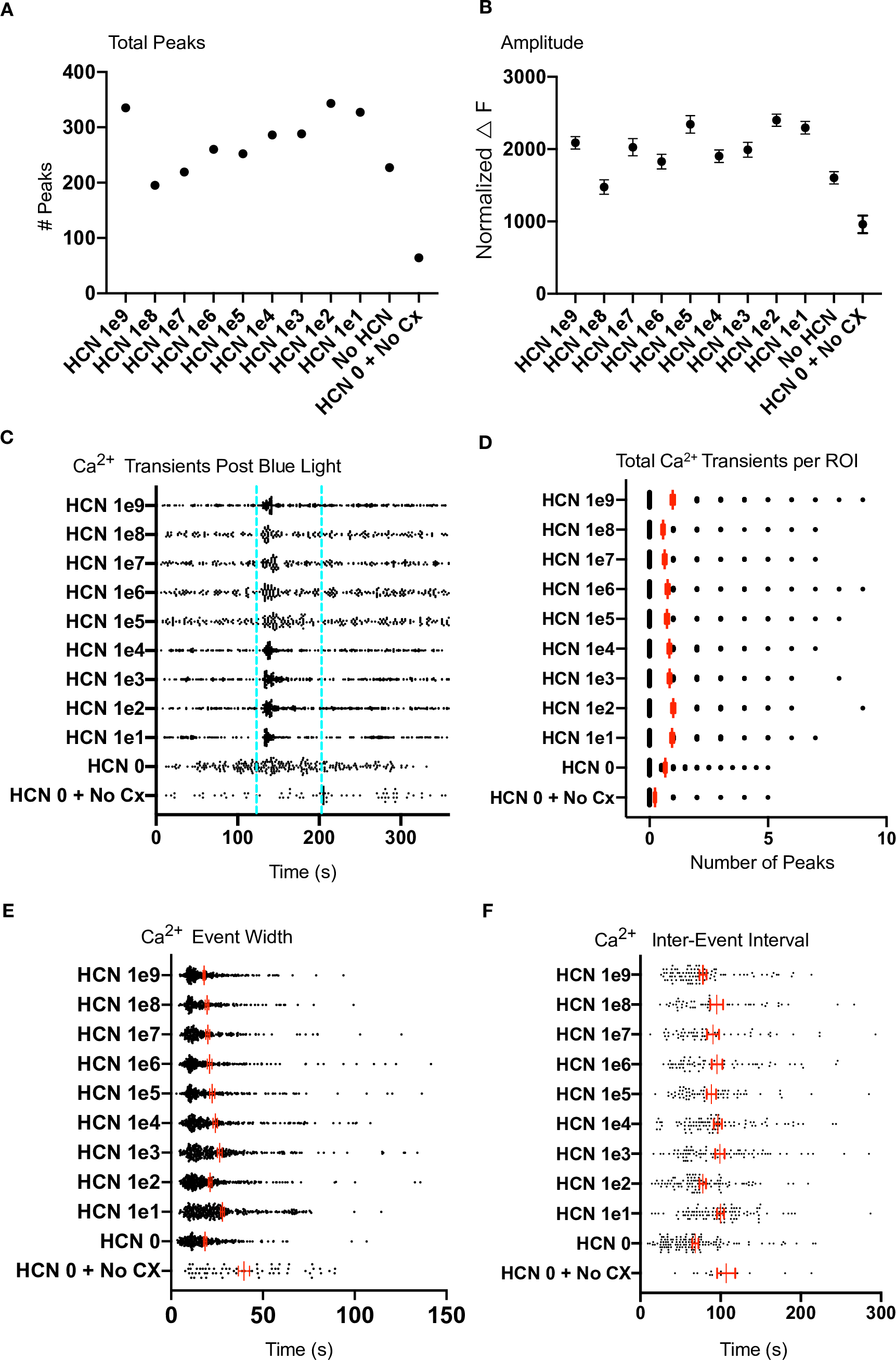

Supplement: S8 Fig — Corresponding S10 Fig. A) The total peaks for each Ca2+ transient within a trial (T = 300s) was recorded. The experiment with [HCN2 1e2] VG/μL had the greatest recorded peaks. B) The △F is the difference between the baseline fluorescence and the maximum fluorescence of each peak. The mean and S.E.M of the Ca2+ transients in HCN2 optimized Kuhl-HKNaCx cells C) Blue line indicates point of 20s blue light stimulus. The time of each fluorescence peak was recorded and is indicated by a black dot. D) The total number of Ca2+ transients per cell (ROI) for experimental HCN2 optimized Kuhl-HKNaCx. E) Representative duration of elevated R-GECO1 fluorescence over time per Ca2+ transient. Black dot indicates the Ca2+ transients FWHM for each peak. F) Black dot indicates the time between Ca2+ transient events per cell. Red bars indicate mean and ± S.E.M (n = 150 cells per condition). (TIF) [file pone.0229051.s008.tif]

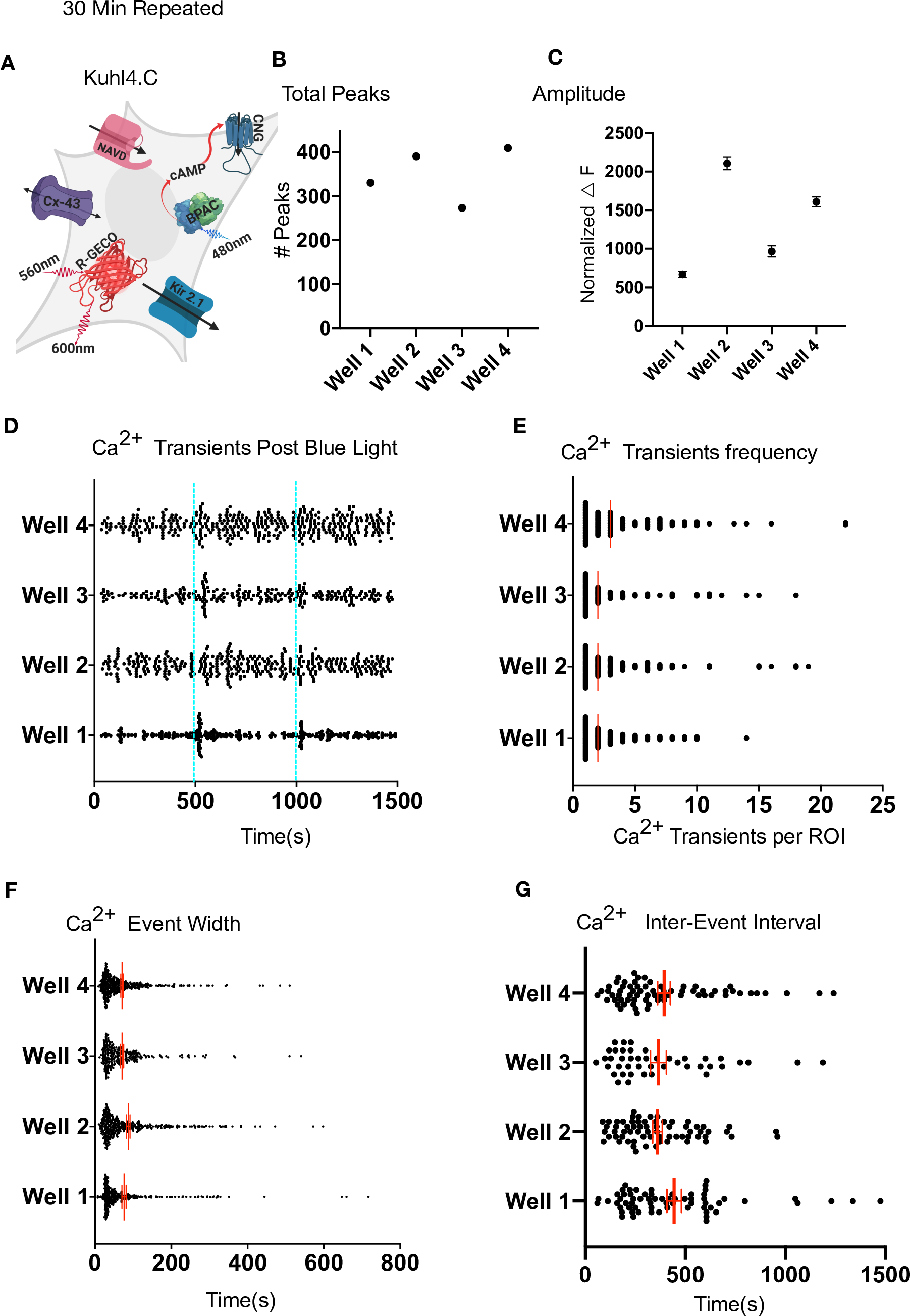

Supplement: S9 Fig — Corresponding S11A Fig. A) The total peaks for each Ca2+ transient within a trial (T = 25m) was recorded. B) The △F is the difference between the baseline fluorescence and the maximum fluorescence of each peak. The mean and S.E.M of the Ca2+ transients in optimized Kuhl-CKNaCx cells C) Blue line indicates point of 20s blue light stimulus. The time of each fluorescence peak was recorded and is indicated by a black dot. D) The total number of Ca2+ transients per cell (ROI) for optimized Kuhl-CKNaCx cells. Representative duration of elevated R-GECO1 fluorescence over time per Ca2+ transient. Black dot indicates the Ca2+ transients FWHM for each peak. F) Black dot indicates the time between Ca2+ transient events per cell. Red bars indicate mean and ± S.E.M (n = 150 cells per condition). (TIF) [file pone.0229051.s009.tif]

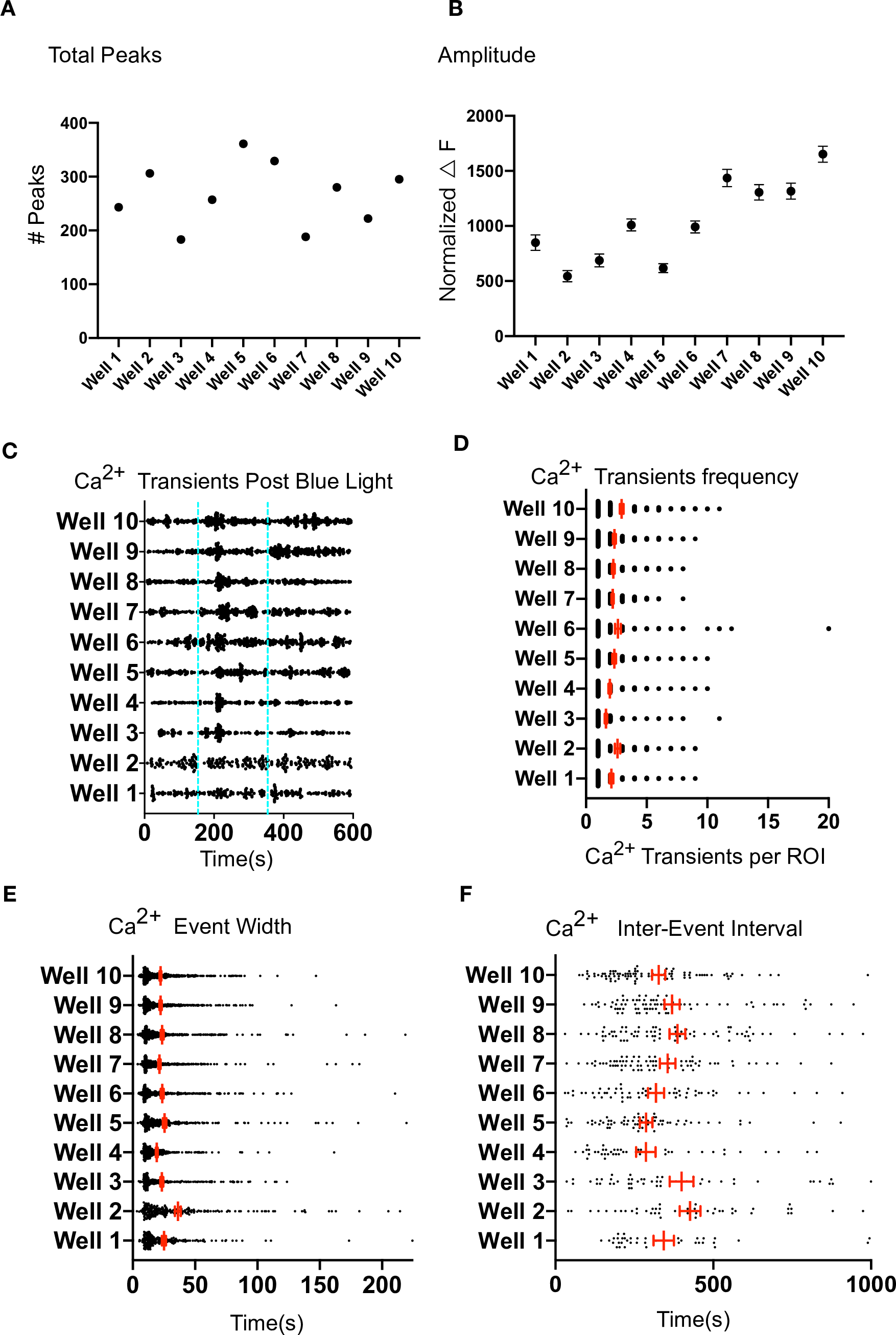

Supplement: S10 Fig — Corresponding S11B Fig. A) The total peaks for each Ca2+ transient within a trial (T = 25m) was recorded. B) The △F is the difference between the baseline fluorescence and the maximum fluorescence of each peak. The mean and S.E.M of the Ca2+ transients in optimized Kuhl-CKNaCx cells C) Blue line indicates point of 20s blue light stimulus. The time of each fluorescence peak was recorded and is indicated by a black dot. D) The total number of Ca2+ transients per cell (ROI) for optimized Kuhl-CKNaCx. Representative duration of elevated R-GECO1 fluorescence over time per Ca2+ transient. Black dot indicates the Ca2+ transients FWHM for each peak. F) Black dot indicates the time between Ca2+ transient events per cell. Red bars indicate mean and ± S.E.M (n = 150 cells per condition). (TIF) [file pone.0229051.s010.tif]

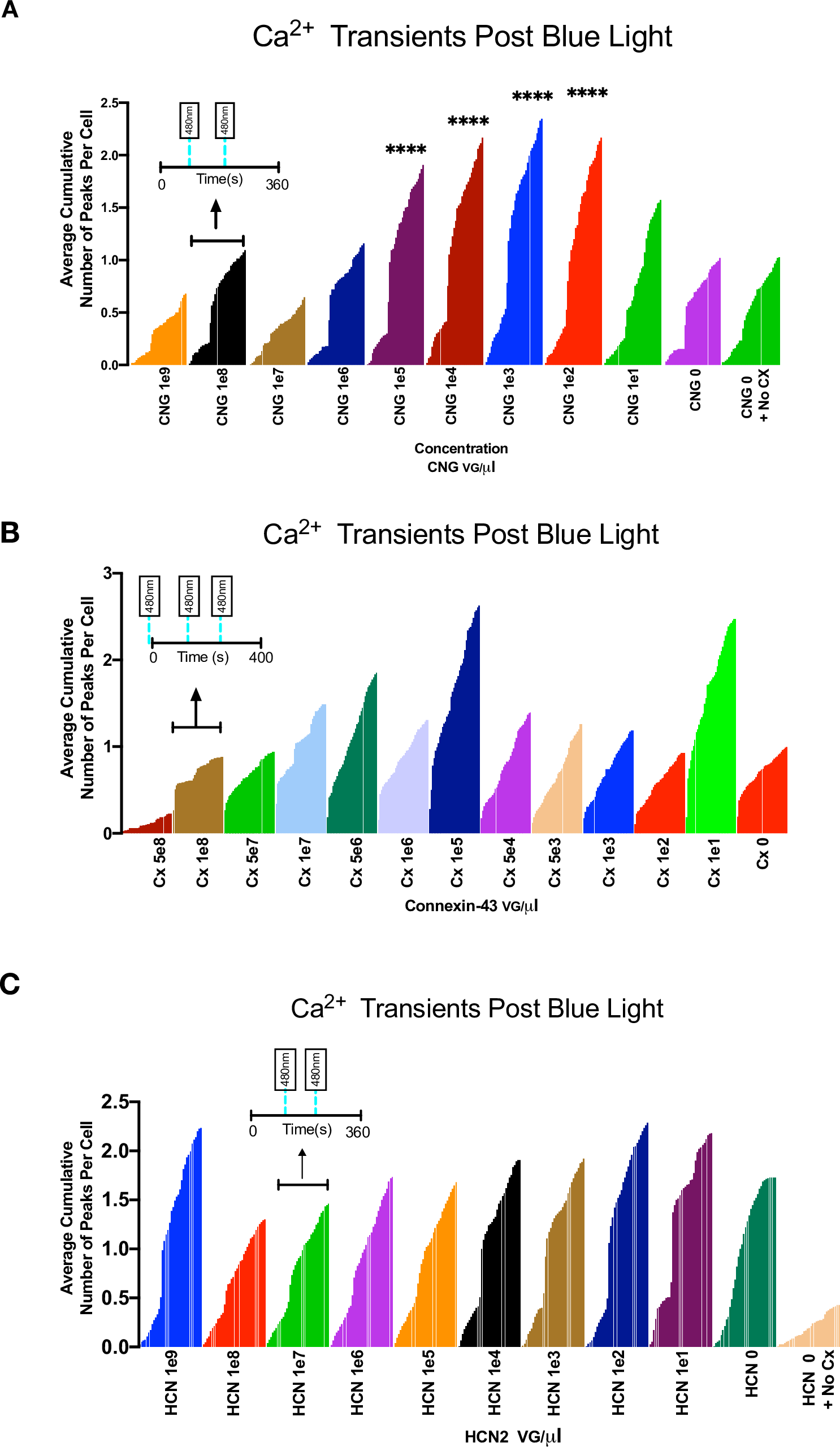

Supplement: S11 Fig — A) Activity was optimized by varying the viral concentration of CNG. Differences were analyzed using Dunnett’s multiple comparison test on the Ca2+ transient frequency (S7D Fig) compared to the control (CNG 0) VG/μl. The experimental trials that were statistically greater than the control were marked (****p ≤ .0001) on the graph. B) The gap junctions between cells using connexin-43 was optimized by varying levels of viral titer. C) Activity was optimized by varying the viral concentration of HCN2. Shown are the average cumulative fluorescence peaks per cell across 360s for each indicated viral concentration of HCN2 (n = 150 cells per condition). (TIF) [file pone.0229051.s011.tif]

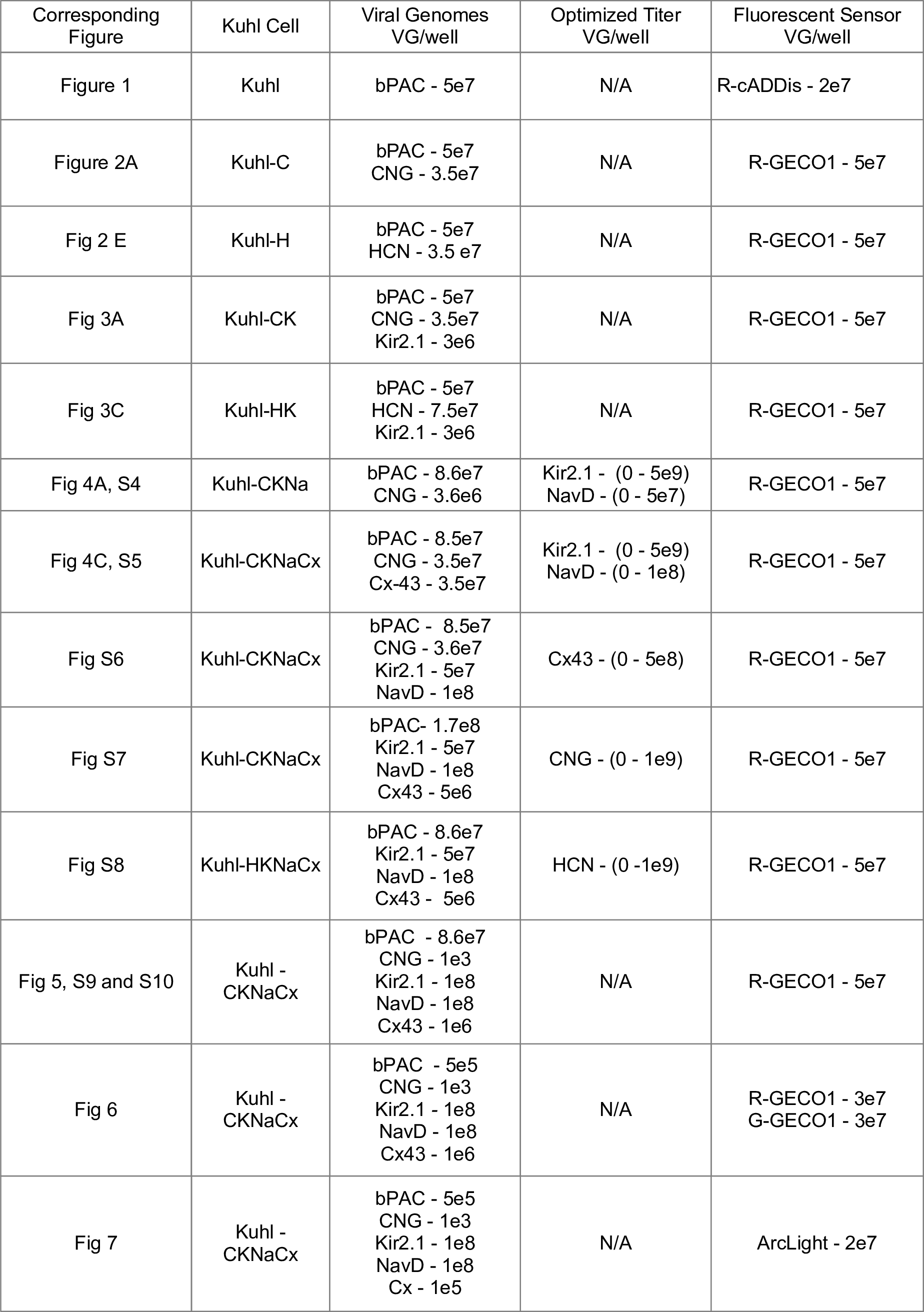

Supplement: S1 Table — The table shows the experimental set up, the final viral concentration used, and their corresponding figures. (TIF) [file pone.0229051.s012.tif]

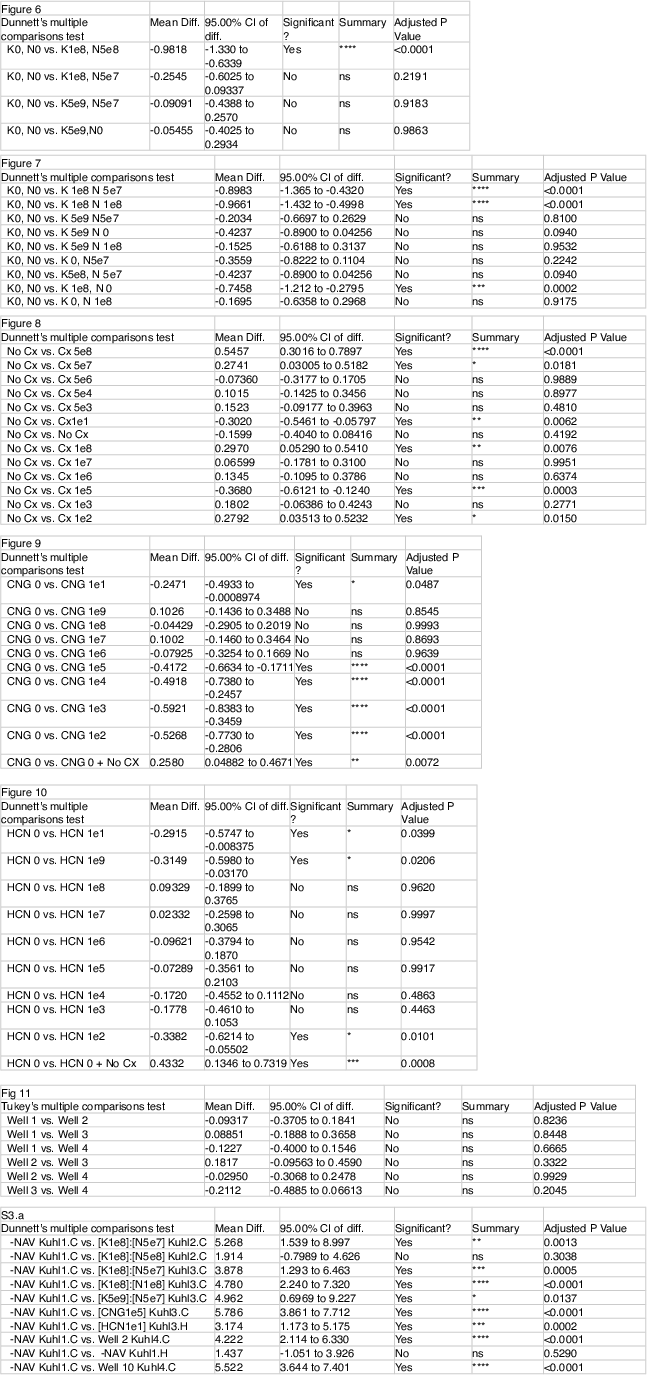

Supplement: S2 Table — The table shows all test run using Dunnetts where stated in the manuscript. (TIF) [file pone.0229051.s013.tif]
